# Phloem iron remodels root development in response to ammonium as the major nitrogen source

**DOI:** 10.1038/s41467-022-28261-4

**Published:** 2022-01-28

**Authors:** Xing Xing Liu, Hai Hua Zhang, Qing Yang Zhu, Jia Yuan Ye, Ya Xin Zhu, Xiang Ting Jing, Wen Xin Du, Miao Zhou, Xian Yong Lin, Shao Jian Zheng, Chong Wei Jin

**Affiliations:** 1 State Key Laboratory of Plant Physiology and Biochemistry, College of Natural Resources and Environmental Science, Zhejiang University, Hangzhou, 310058 China; 2 State Key Laboratory of Plant Physiology and Biochemistry, College of Life Science, Zhejiang University, Hangzhou, 310058 China

**Keywords:** Plant physiology, Abiotic, Plant development

## Abstract

Plants use nitrate and ammonium as major nitrogen (N) sources, each affecting root development through different mechanisms. However, the exact signaling pathways involved in root development are poorly understood. Here, we show that, in *Arabidopsis thaliana*, either disruption of the cell wall-localized ferroxidase LPR2 or a decrease in iron supplementation efficiently alleviates the growth inhibition of primary roots in response to NH_4_^+^ as the N source. Further study revealed that, compared with nitrate, ammonium led to excess iron accumulation in the apoplast of phloem in an LPR2-dependent manner. Such an aberrant iron accumulation subsequently causes massive callose deposition in the phloem from a resulting burst of reactive oxygen species, which impairs the function of the phloem. Therefore, ammonium attenuates primary root development by insufficiently allocating sucrose to the growth zone. Our results link phloem iron to root morphology in response to environmental cues.

## Abstract (web summary)

Ammonium affects plant root development through different mechanisms than nitrate. Here, the authors show that the Arabidopsis cell wall-localized ferroxidase LPR2 is required to attenuate root growth in response to ammonium.

## Introduction

Nitrogen (N) is the essential nutrient required in the greatest quantity by plants. It is primarily acquired by plant roots in the forms of ammonium (NH_4_^+^) and nitrate (NO_3_^−^). Nevertheless, NH_4_^+^ is notoriously toxic to most plants when present as the only or predominant N source^1^, which is not the case for NO_3_^−^. In fact, NH_4_^+^ stress is of major importance to the ecology of soils that exhibit higher ammonification than nitrification rates, such as those found in wetlands, including saltmarshes, bogs, mangroves, and fens^2–4^. In addition, because of highly intensive tillage or waterlogged cultivation methods, many crop plants also suffer long-term NH_4_^+^ stress induced by the over-application of N fertilizers^5^. Identification of the mechanisms underlying toxicity and detoxification of NH_4_^+^ in plants is crucial for improving the adaptation of the plants in the presence of NH_4_^+^ as the primary N source.

Similar to many other abiotic cues, NH_4_^+^ stress also inhibits the growth of primary roots (PRs); hence, stunted PR growth is recognized as a typical symptom of NH_4_^+^ toxicity^6,7^. The uptake of NH_4_^+^ by roots is the first step affecting cellular NH_4_^+^ concentration and therefore is an important process affecting NH_4_^+^ stress in plants. Knocking out NH_4_^+^ transporters (AMTs) AtAMT1;1, AtAMT1;2, and AtAMT1;3 results in a 90% reduction in NH_4_^+^ uptake and substantially decreases NH_4_^+^ sensitivity^8^. The protein kinase AtCIPK23 phosphorylates and inactivates AtAMT1;1 and AtAMT1;2, and thus activation of *AtCIPK23* expression by transcription factor AtSTOP1 has been identified as a mechanism for NH_4_^+^ tolerance^9,10^. A decrease in NH_4_^+^ uptake due to enhanced proton extrusion from the resulting activation of H^+^-ATPase has also been shown to be a mechanism for NH_4_^+^ tolerance^11,12^.

A high concentration of cellular NH_4_^+^ also causes a variety of physiological damage in plants, such as disordered pH regulation, excess energy consumption, deficiency of mineral cations, increased oxidative stress, and disruption of hormonal homeostasis^4,6,13^. Lines of evidence have linked some of these effects to NH_4_^+^ toxicity. For example, the reaction of the glutamine synthetase GLN2 in shoots over-produces protons, resulting in pH disorder and consequently contributing to NH_4_^+^ toxicity in the shoots^14^; NH_4_^+^ stress results in excessive production of hormone ethylene, while EIN2- and EIN3-mediated ethylene signaling partly causes NH_4_^+^ toxicity in shoots^15,16^. However, these physiological effects are indirectly related, and most of them explain NH_4_^+^ toxicity in shoots but not in the roots^6^.

Currently, only a few signaling events have been proposed to mediate NH_4_^+^-induced PR growth inhibition. A previous study showed that knockout of *VTC1*, which encodes a GDP-mannose pyrophosphorylase, results in greater inhibition of PR growth under NH_4_^+^ stress, but the underlying mechanism remains unclear^17^. Auxin was also suggested to be associated with the NH_4_^+^-sensitive phenotype of PRs^18^. However, it has been shown that the PR growth of auxin transport mutant *aux1* is similar to that of the wild-type plant under NH_4_^+^ stress^19^, leaving it open as to whether auxin plays a role in NH_4_^+^-induced PR growth inhibition. Therefore, the mechanisms, particularly the molecular pathways, underlying the negative impacts of NH_4_^+^ in PR growth remain largely unknown^4,6^.

In this work, we screened an Arabidopsis (*Arabidopsis thaliana*) transfer DNA (T-DNA) insertion mutant whose PR growth is insensitive to NH_4_^+^ stress. The T-DNA insertion in this mutant was annotated in the gene *LOW PHOSPHATE ROOT 2* (*LPR2*), which encodes a ferroxidase. Since the biological processes of roots might be artificially affected by photo-Fenton reactions resulting from the light-irradiated rooting conditions^20,21^, a shaded rooting system was used in our study. We further identified a mechanism that connects remodeling of root development to NH_4_^+^ stress: the NH_4_^+^ induces massive Fe accumulation in phloem via the action of LPR2, and as a result, arrests PR development due to the inhibition of phloem function.

## Results

### The NH_4_^+^-sensitive phenotype of PRs depends on the action of LPR2

An NH_4_^+^-insensitive Arabidopsis mutant *isas* was initially screened from a T-DNA insertion library^22^ using transparent Petri dishes with a light-irradiated rooting medium. This mutant was found to be the SALK_056696 line, with the T-DNA insertion in the second exon of *LPR2* (AT1G71040) (Supplementary Fig. 1a, b). qRT-PCR and immunoblot assay showed that *isas* is most probably a *LPR2* knockout mutant (Supplementary Fig. 1c, d). As natural soils normally have pH-buffering capacity, the pH buffer MES was added to the growth media in our study, unless specified otherwise. When grown on a control medium with NO_3_^−^ as the N source, the *isas* roots did not differ morphologically from wild-type Col-0 roots. In the medium with NH_4_^+^ as the N source, however, the *isas* mutants showed better PR growth than Col-0 seedlings did (Supplementary Fig. 2).

**Fig. 1:**
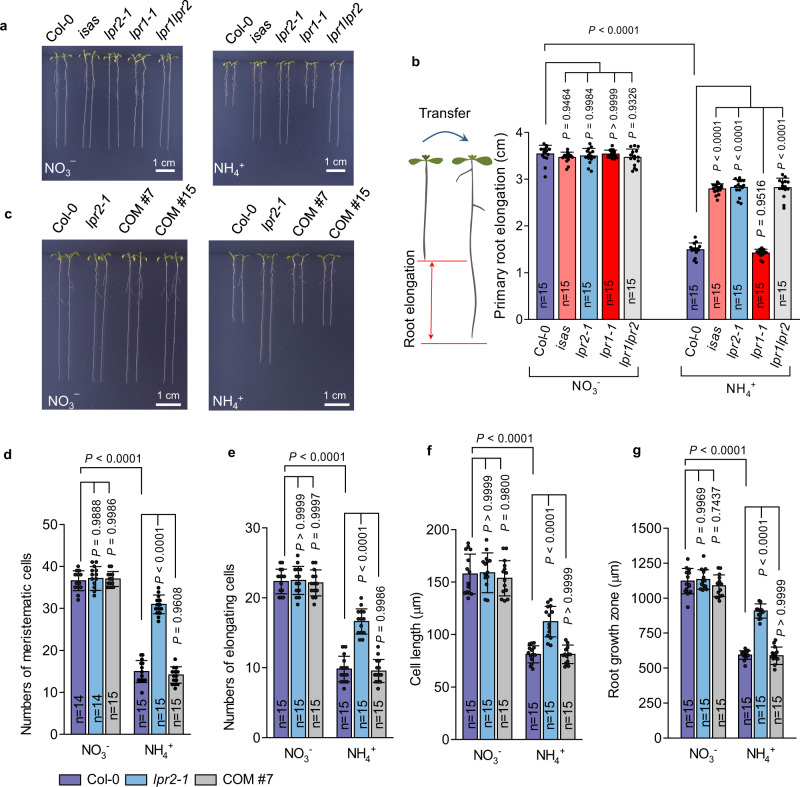
LPR2-dependent growth inhibition of primary roots in NH4+ medium. **a** Phenotype comparison of Col-0 and the *isas*, *lpr2-1*, *lpr1-1*, and *lpr1lpr2* mutants. **b** Elongation of primary roots. **c** Phenotype comparison of Col-0, *lpr2-1*, and complementation lines (COM #7 and COM #15). **d** Number of meristematic cells. **e** Number of elongation cells. **f** Length of the first differentiated cell. **g** Length of the root growth zone (elongation plus meristem zone). **b**, **d**-**g** Data shown are mean ± SD. *P* values < 0.05 indicate significant differences (two-way ANOVA with post-hoc Tukey HSD test; *n* = number of seedlings). Four-day-old seedlings of the indicated genotypes were transferred to NO_3_^−^ or NH_4_^+^ medium with 100 µM Fe and analyzed 4 days after seedling transfer. Each experiment was repeated independently at least three times with similar results, and a representative experiment is shown.

Unless specified otherwise, we used a black plastic sheet to cover the roots to exclude light irradiation in the remaining studies (Supplementary Fig. 3). Under such root-shaded conditions, the *isas* mutants still had greater PR elongation in the NH_4_^+^-N source than Col-0 seedlings did (Fig. 1a, b). In addition, another T-DNA insertion mutant, *lpr2-1* (SALK_091930)^23^, which is also probably a knockout mutant (Supplementary Fig. 1), had a similar NH_4_^+^-insensitive phenotype. The NH_4_^+^ sensitivities of both *lpr2* mutants were also examined in unbuffered conditions, and both mutants consistently had better PR growth than Col-0 seedlings did in the NH_4_^+^-N source without MES (Supplementary Fig. 4). These results show that the inhibitory effect of NH_4_^+^ on PR growth could be uncoupled by disruption of LPR2.

LPR1 (AT1G23010) is a close paralog of LPR2^23^. However, the NH_4_^+^-sensitive PR phenotype of the *lpr1-1* mutant did not differ from that of Col-0 seedlings (Fig. 1a, b). Moreover, the *lpr1lpr2* double mutants did not have greater insensitivity to NH_4_^+^ than *lpr2* single mutants did. Accordingly, the NH_4_^+^ sensitivity of PRs was only associated with *LPR2* and not with *LPR1*. We also generated *pLPR2::LPR2-YFP* transformants in the *lpr2-1* background. These complementation lines (COM#7 and COM#15) had moderately higher *LPR2* expression and LPR2 abundance than Col-0 seedlings did (Supplementary Fig. 5) and fully restored the inhibitory effect of NH_4_^+^ on PR growth (Fig. 1c). These results further demonstrate that *LPR2* is responsible for the NH_4_^+^ sensitivity of PRs.

Root elongation is controlled by cell division and differentiation along the longitudinal axis of the root^24^. Here, we showed that the meristematic cell numbers in PRs of Col-0 and COM#7 seedlings in the NH_4_^+^-N source were both decreased by ~60% compared with those in the NO_3_^−^-N source, whereas only a slight decrease was observed in *lpr2-1* mutants (Fig. 1d). This indicates that LPR2 acts to accelerate a shift from cell division to differentiation. In addition, both the elongation cell number and the first differentiated cell length in PRs of Col-0 and COM#7 seedlings were also greatly reduced by NH_4_^+^, and such reductions were clearly diminished in the *lpr2-1* PRs (Fig. 1e, f). As a result, in the NH_4_^+^, the *lpr2-1* mutants had less growth stunting in the root growth zone (meristem plus elongation zone) than Col-0 and COM#7 seedlings did (Fig. 1g). Therefore, the PR growth inhibition resulting from NH_4_^+^ stress highly depends on LPR2 and is initiated by an accelerated shift from cell division to differentiation in the meristem zone, followed by early differentiation of elongating cells.

### LPR2 encodes a cell wall ferroxidase and is irreplaceable by LPR1

**Fig. 2:**
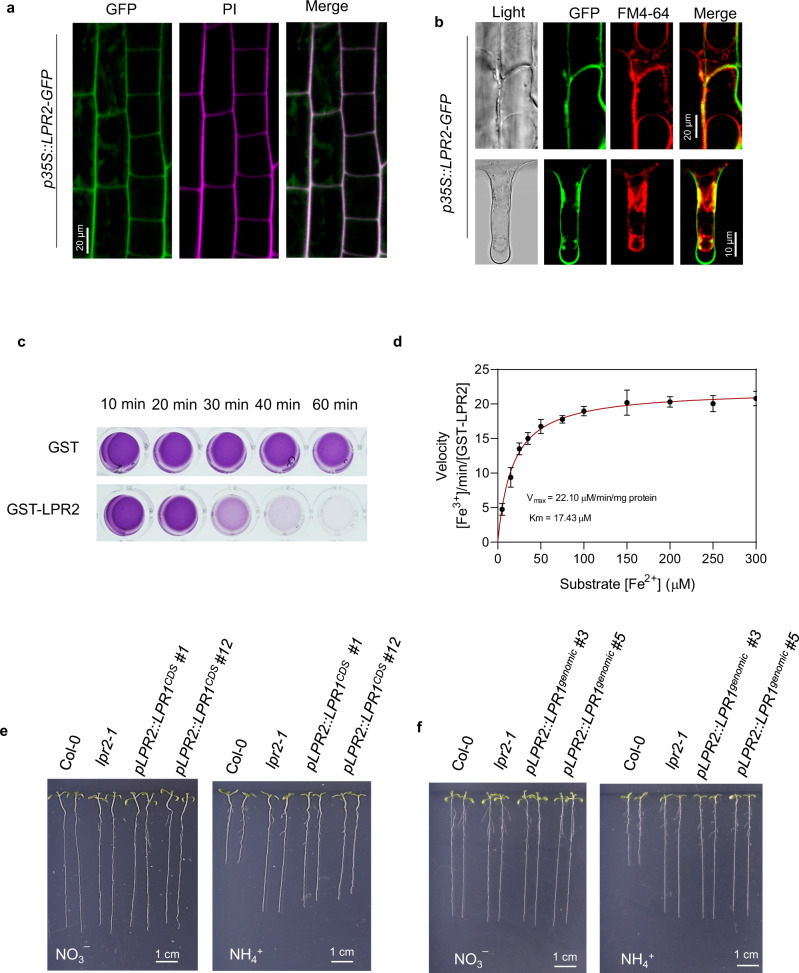
LPR2 is a cell wall ferroxidase and is irreplaceable by LPR1. **a** Fluorescence co-localization of LPR2-GFP and propidium iodide in cells of *p35S::LPR2-GFP* roots. **b** Fluorescence of LPR2-GFP and FM4-64 in cells of *p35S::LPR2-GFP* roots after plasmolysis with 0.8 M sorbitol. Four-day-old seedlings were transferred to NH_4_^+^ medium with 100 µM Fe, and confocal analyses were performed 4 days after seedling transfer. **c** Ferroxidase activities of recombinant GST-LPR2. Ferroxidase assay using 1 μg purified GST-LPR2 protein. Pink indicates the Fe^2+^-ferrozine complex. The substrate Fe^2+^ was added in the form of Fe(NH_4_)_2_(SO_4_)_2_·6H_2_O at an initial concentration of 50 μM. **d** Fe^2+^ concentration-dependent (0–300 μM) ferroxidase activity of GST-LPR2 protein. Data shown are mean ± SD of three biological replicates. **e**, **f** Phenotype of *lpr2-1* mutant lines with LPR2 promoter-confined tissue-specific expression of *LPR1*^*CDS*^ and *LPR1*^*genomic*^. Four-day-old seedlings of the indicated genotypes were transferred to NO_3_^−^ or NH_4_^+^ medium with 100 µM Fe and analyzed 4 days after seedling transfer. Two (**c**, **d**) or three (**a**, **b**, **e**, **f**) independent experiments were performed with similar results, and one representative experiment is shown.

Prediction by the SignalP 5.0 algorithm^25^ showed that both LPR1 and LPR2 contained a putative secretory signal peptide at the N-terminal region (Supplementary Fig. 6). Consistent with this prediction, confocal analysis of *p35S::LPR2-GFP* transformants revealed that LPR2-GFP fluorescence mainly resided in the cell wall matrix (Fig. 2a, b), which is very similar to the subcellular localization of LPR1^26^. LPR1 was previously characterized to have ferroxidase activity that converts Fe^2+^ to Fe^3+,^^26,27^. As expected, the recombinant GST-LPR2 protein from *Escherichia coli* also had obvious ferroxidase activity, and exhibited a typical Michaelis–Menten kinetic with a K_m_ and V_max_ of 17.43 µM and 22.10 µM/min/mg protein, respectively (Fig. 2c, d).

Considering the similarities of LPR2 and LPR1 in both subcellular localization and enzymatic reaction, we explored whether the role of LPR2 in mediating NH_4_^+^ sensitivity could be filled by LPR1. We used the *LPR2* promoter to confine the site of *LPR1* expression in the *lpr2-1* mutant. However, although both *pLPR2::LPR1*^*CDS*^/*lpr2-1* and *pLPR2::LPR1*^*genomic*^/*lpr2-1* transformants had comparable or slightly higher expression levels of *LPR1* compared with that of *LPR2* in Col-0 seedlings (Supplementary Fig. 7), they still showed similar PR growth as that of the *lpr2-1* seedlings in the NH_4_^+^-N source (Fig. 2e, f). Therefore, LPR2 is irreplaceable by LPR1 in mediating NH_4_^+^ sensitivity, which may explain why only LPR2 and not LPR1 acts to suppress PR growth in the presence of NH_4_^+^.

### The LPR2-mediated NH_4_^+^-sensitive PR phenotype depends on the action of Fe

**Fig. 3:**
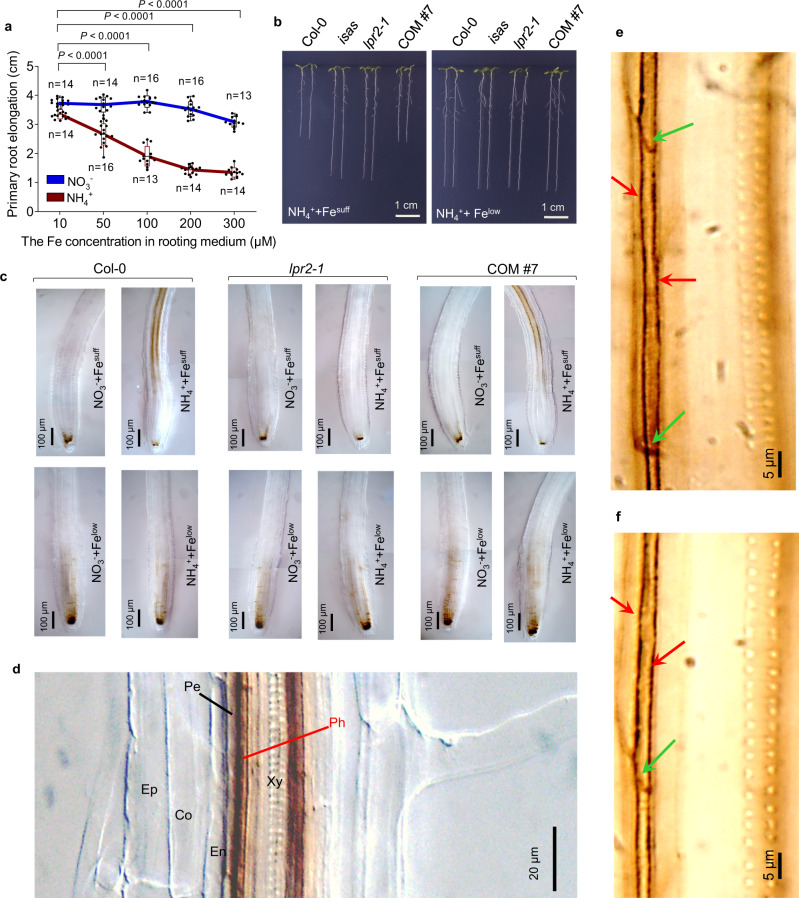
Fe-dependent inhibition of primary root growth in NH4+ medium. **a** Primary root elongation of Col-0 seedlings in NO_3_^−^-N or NH_4_^+^-N source with various doses of Fe. Center line represents mean and bounds of box are SD; whiskers indicate the minimum and maximum values; *n* = number of seedlings. *P* values <0.05 indicate significant interactions between N form and Fe dose (two-way ANOVA with post-hoc Tukey HSD test). **b** NH_4_^+^-sensitivity comparison of Col-0, *isas*, *lpr2-1*, and complementation line COM#7 in Fe^suff^ (100 µM) and Fe^low^ (10 µM) conditions. **c** Fe deposition indicated by Perls/DAB staining in primary roots. **d** Close-up view of Fe deposition in root stele of Col-0 seedlings grown in Fe^suff^ NH_4_^+^ medium. Ep epidermis, Co cortex, En endodermis, Pe pericycle, Ph phloem, Xy xylem. **e** Perls/DAB staining in the phloem of the primary roots of Col-0 seedlings grown in Fe^suff^ NH_4_^+^ medium. Red and green arrows show Fe depositions at lateral cell walls and sieve plates of the phloem, respectively. **f** Acetone washed-Perls/DAB staining in phloem of the primary roots of Col-0 seedlings grown in Fe^suff^ NH_4_^+^ medium. The Perls/DAB-stained roots were washed with acetone for 3 h. Four-day-old seedlings of the indicated genotypes were transferred to NO_3_^−^ or NH_4_^+^ medium with various doses of Fe supply and analyzed 4 days after seedling transfer. Each experiment was repeated independently three times with similar results, and a representative experiment is shown.

We next asked how LPR2 mediates the NH_4_^+^ sensitivity of PRs. Disruption of LPR2 did not affect the NH_4_^+^ level in roots (Supplementary Fig. 8), showing that LPR2-mediated NH_4_^+^ sensitivity is not associated with NH_4_^+^ accumulation. Since LPR2 is a ferroxidase, the role of Fe in regulating NH_4_^+^ sensitivity of PRs was investigated. We first grew Arabidopsis seedlings in media with different doses of Fe. Fe doses ≤10 µM ensured a similar PR growth of Col-0 between NH_4_^+^- and NO_3_^−^-N sources, whereas ≥50 µM induced a clear dose-dependent inhibition of PR growth in the NH_4_^+^-N source (Supplementary Fig. 9 and Fig. 3a). In contrast, supplementing deferoxamine or ferrozine, two potent Fe chelators, almost completely rescued PR growth of Col-0 seedlings in the NH_4_^+^-N source with 100 µM Fe (Supplementary Fig. 10). The inhibitory effect of NH_4_^+^ on root growth of rice plants was also uncoupled by lowering Fe supply (Supplementary Fig. 11). These results indicate that the NH_4_^+^ sensitivity of roots strongly depends on the action of Fe.

Given that Fe-free (0 µM) and high-Fe (≥200 µM) conditions may respectively cause Fe deficiency and Fe excess in Arabidopsis, we used 10 and 100 µM Fe, defined as low Fe (Fe^low^) and sufficient Fe (Fe^suff^), respectively, in the remaining evaluations. The difference in NH_4_^+^ sensitivity between *lpr2* mutants and either Col-0 or COM#7 seedlings was completely abolished when the Fe concentration was lowered from Fe^suff^ to Fe^low^ (Fig. 3b), showing that the LPR2-mediated NH_4_^+^-sensitive phenotype depends on the action of Fe.

Furthermore, we tested whether Fe plays a role in NH_4_^+^ sensitivity of PRs when the N source has a high NH_4_^+^/NO_3_^−^ ratio (10:2). Similarly, the difference in PR growth inhibition between *lpr2* mutants and Col-0 seedlings at a high NH_4_^+^/NO_3_^−^ ratio was also abolished by lowering Fe supplement (Supplementary Fig. 12). Therefore, Fe is required for the remolding of PR development when NH_4_^+^ is the only or predominant N source.

### NH_4_^+^ induces Fe deposition in the phloem

The strong dependence of the NH_4_^+^-sensitive PR phenotype on Fe raised the question of whether NH_4_^+^ stress affects Fe deposition in roots. We used an Fe-specific histochemical procedure, Perls/3,3’-diaminobenzidine (DAB) staining^28^, to visualize Fe distribution in PRs. For Col-0 and *lpr1-1* seedlings with Fe^suff^, the NH_4_^+^-N source resulted in massive Fe deposition in root stele compared to that of the NO_3_^−^-N source (Fig. 3c and Supplementary Fig. 13a). In contrast, the NH_4_^+^-induced Fe deposition was completely abolished by either disruption of LPR2 in *lpr2-1* mutants or Fe^low^ treatment in Col-0 seedlings. The complementation line COM#7 restored the Fe deposition in root stele in the NH_4_^+^-N source with Fe^suff^. These results indicate that NH_4_^+^-induced Fe deposition in root stele depends on LPR2 as well as on a sufficient Fe supply. Notably, these respective Fe depositions across the various genotypes and Fe-dose treatments were negatively correlated with PR growth in the NH_4_^+^-N source (Fig. 3b), suggesting that the Fe deposition in root stele determines the PR growth response to NH_4_^+^ stress. Surprisingly, in the presence of either NO_3_^−^ or NH_4_^+^, Fe^low^ treatment resulted in a higher Fe deposition in the root stem cell niche (SCN) of Col-0, *lpr2-1*, and COM#7 seedlings compared with that with Fe^suff^ treatment (Fig. 3c). Nevertheless, although no Fe deposition was detected in the SCN of *lpr1lpr2* seedlings (Supplementary Fig. 13b), this double mutant displayed similar NH_4_^+^ insensitivity as that of the *lpr2-1* mutant (Fig. 1c), indicating that the NH_4_^+^ sensitivity of PRs is independent of the Fe deposition in SCN.

A close-up view of Perls/DAB-stained Col-0 roots from Fe^suff^ NH_4_^+^ treatment showed that the greatest Fe deposition was in phloem cells (Fig. 3d). At higher magnification, Fe was found to be mainly located at the periphery of sieve elements (Fig. 3e). This periphery is probably the apoplast of phloem, because the LPR2 responsible for this Fe deposition is a cell wall-resident protein. Since the phloem vasculature was difficult to plasmolyze using the methods of osmotic dehydration, we used acetone to wash off the plasmalemma. This procedure did not alter Fe deposition at the periphery of sieve elements (Fig. 3f), providing further support for apoplastic Fe deposition in the phloem.

Unexpectedly, the NH_4_^+^-N source with Fe^suff^ resulted in heavy Fe deposition in all cell types of Col-0 PRs under light-irradiated rooting conditions, which was significantly different from that observed under root-shaded conditions (Supplementary Fig. 14). The result strongly suggests that the impact of artificial light irradiation on roots cannot be neglected in studying the mechanisms of root growth responses to NH_4_^+^, even though root growth inhibition by NH_4_^+^ is barely affected by the light irradiation on roots.

### LPR2 distributes in root stele and is upregulated by NH_4_^+^

**Fig. 4:**
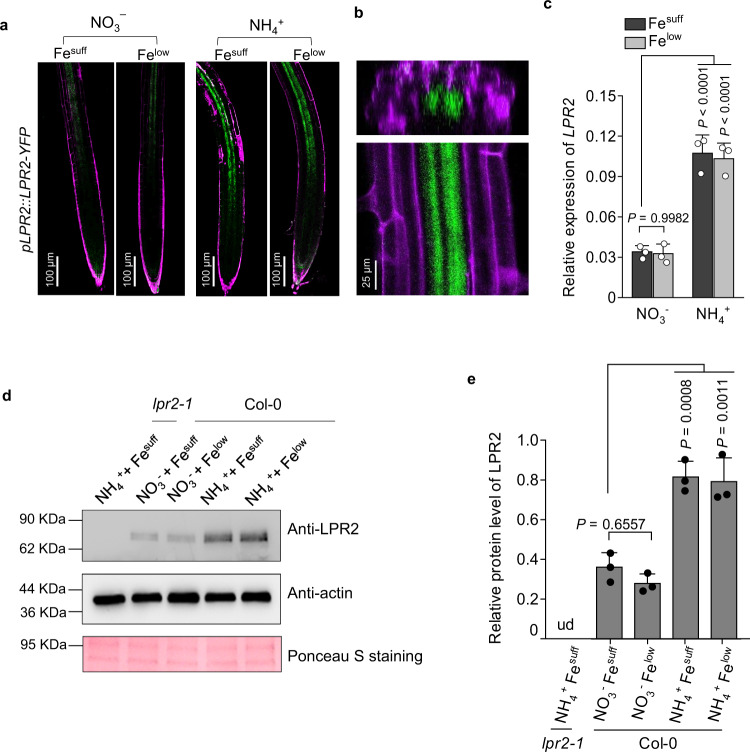
Distribution of LPR2 in roots and its response to NH4+. **a** *pLPR2::LPR2-YFP* expression in primary roots of complementation line COM#7. Roots were counterstained with propidium iodide (purple fluorescence) and analyzed for YFP fluorescence (green). **b** Radial section (top) and close-up view (bottom) of *pLPR2::LPR2-YFP* expression in a root of a COM#7 seedling grown in Fe^suff^ NH_4_^+^ medium. **c** *LPR2* expression in Col-0 roots. Relative expression levels were normalized to the geometric mean of expression of *UBQ10* and *EF1α*. **d**, **e** Representative gels and relative protein levels in Col-0 roots. Relative LPR2 levels were estimated from the ratio of the signal intensity of LPR2 to that of actin from the same sample. **c**, **e** Data shown are mean ± SD of three biological replicates. *P* values < 0.05 indicate significant differences (two-way ANOVA with post-hoc Tukey HSD test). Four-day-old seedlings of the indicated genotypes were transferred to NO_3_^−^ or NH_4_^+^ medium with Fe^suff^ (100 µM) or Fe^low^ (10 µM) and analyzed 4 days after seedling transfer. Each experiment was repeated independently three times with similar results, and a representative experiment is shown.

The NH_4_^+^-induced phloem-specific Fe deposition prompted us to analyze the tissue distribution of LPR2 in PRs of *pLPR2::LPR2-YFP/lpr2-1* transformants. LPR2-YFP was mainly distributed in the root stele, but its abundance was lower in the NO_3_^−^-N source. LPR2-YFP fluorescence was increased in the NH_4_^+^-N source, while the dose of Fe had little effect on this response (Fig. 4a). Unexpectedly, LPR2 was found to be ubiquitously distributed in the whole root stele except in the xylem (Fig. 4b), an area much larger than the Fe deposition region (i.e., phloem). This suggests that other factor(s) are required for phloem-specific Fe deposition and NH_4_^+^-induced PR growth inhibition. Unfortunately, we were unable to identify the relative factor(s) in the present study.

LPR1 distribution in PRs was also examined in *pLPR1::LPR1-YFP*/*lpr1-1* transformants. The abundance of LPR1-YFP was lower in the presence of either N source, but it was mainly distributed in the SCN of the root apical meristem and slightly expanded to endodermal cell layers (Supplementary Fig. 15). The difference in tissue distribution may be another part of the explanation of why LPR1 does not play a role in mediating the NH_4_^+^-sensitive phenotype as LPR2 does.

The response of LPR2 in Col-0 roots to NH_4_^+^ was also examined. Consistent with the above confocal result, either the *LPR2* expression or the LPR2 abundance in Col-0 roots showed an increase under NH_4_^+^ relative to that under NO_3_^−^, which was also irrespective of the Fe supplementation dose (Fig. 4c–e). Therefore, NH_4_^+^ rather than Fe upregulates LPR2. Interestingly, although the *LPR2* expression in *p35S::LPR2-GFP* overexpression lines was similar between two N sources, the LPR2-GFP abundance in the NH_4_^+^-N source was higher than that in the NO_3_^−^-N source (Supplementary Fig. 16), indicating a post-transcriptional regulation for LPR2. It is worth noting that, although LPR2 was slightly overexpressed in *pLPR2::LPR2-YFP/lpr2-1* complementation lines (Supplementary Fig. 5), their PRs had a similar NH_4_^+^-sensitive phenotype as that of Col-0 seedlings (Fig. 1d). Furthermore, the PRs of *p35S::LPR2-GFP* overexpression lines also had a similar NH_4_^+^ sensitivity as that of Col-0 PRs (Supplementary Fig. 17a). These results indicate that the upregulated portion of LPR2 makes little contribution to NH_4_^+^ sensitivity and further support that, although LPR2 is essential, additional factor(s) are also required for NH_4_^+^-induced PR growth inhibition. It is worth noting that the *p35S::LPR2-GFP* overexpression lines showed irregular Fe deposition in PRs in the NH_4_^+^-N source (Supplementary Fig. 17b), indicating that NH_4_^+^-induced phloem-specific Fe deposition depends on the stele-specific distribution of LPR2.

### NH_4_^+^-induced Fe deposition triggers a burst of reactive oxygen species (ROS)

To understand how LPR2-mediated Fe deposition inhibits PR growth in the NH_4_^+^-N source, we performed a whole transcriptome sequencing (RNA-seq) analysis of gene expression in the roots of Col-0 and *lpr2-1* seedlings (Supplementary Data 1a). The reliability of RNA-seq data was confirmed by qRT-PCR assay for the expression of ten randomly selected genes with different abundances (Supplementary Fig. 18). As Fe^low^ treatment and *LPR2* disruption similarly alleviated PR growth inhibition in the NH_4_^+^-N source, we screened differentially expressed genes (DEGs) (−1 > log_2_[fold change] > 1; *P* value <0.05) by performing pairwise comparisons of transcript abundances either between the two Fe treatments or between *lpr2-1* and Col-0 with Fe^suff^ treatment (Supplementary Data 1b–e). In the NO_3_^−^-N source, there were only 11 DEGs between the two Fe treatments for Col-0 seedlings and 87 DEGs between the two genotypes that received Fe^suff^ treatment (Supplementary Fig. 19a, b). These DEGs are unlikely related to PR development because either Fe^low^ treatment or *LPR2* disruption had little effect on PR growth in NO_3_^−^-N source.

**Fig. 5:**
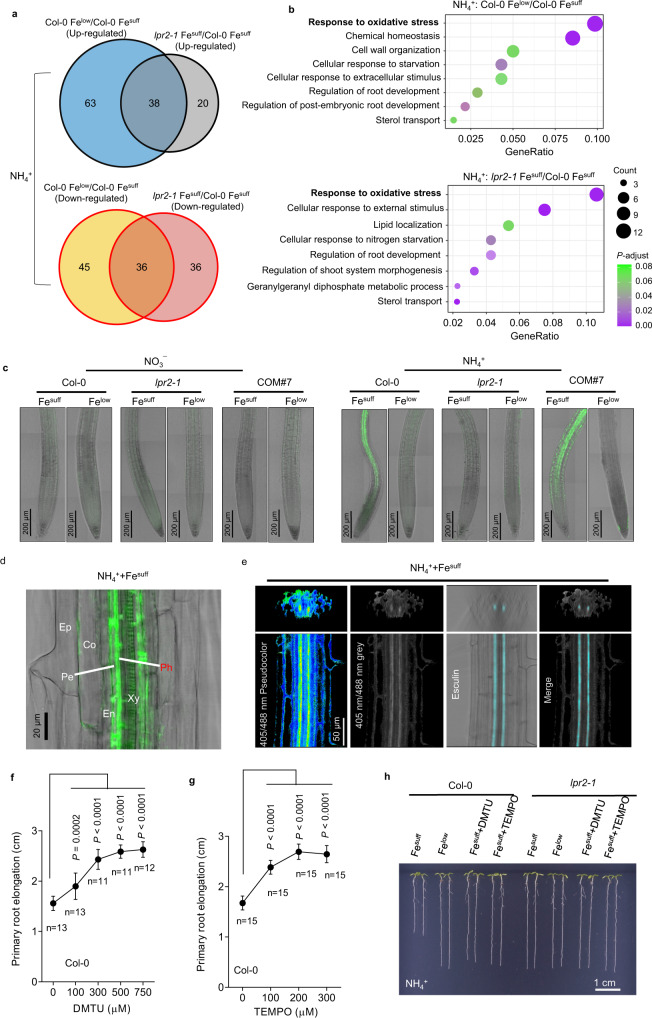
Fe-dependent primary root growth inhibition by NH4+ is associated with a burst in reactive oxygen species (ROS). **a**, **b** Venn diagram and Gene Ontology enrichment analysis of differentially expressed genes (DEGs) that are up- and downregulated in the pairwise comparisons of Col-0 Fe^low^ vs. Col-0 Fe^suff^ and *lpr2-1* Fe^suff^ vs. Col-0 Fe^suff^ in the NH_4_^+^-N source. The size of the circle represents gene numbers, and the color represents the value of *P*-adjust the was calculated by hypergeometric tests and adjusted for multiple testing using FDR. RNA sequencing was conducted with three biological replicates per line and condition. **c** ROS visualization in primary roots of Col-0, *lpr2-1*, and complementation line COM#7 seedlings by H_2_DCFDA staining. **d** Close-up view of H_2_DCFDA staining in primary roots of Col-0 seedlings grown in Fe^suff^ NH_4_^+^ medium. **e** Co-localization of false color representation of H_2_O_2_ and fluorescence of phloem marker esculin in primary roots of roGFP2-Orp1 seedlings grown in Fe^suff^ NH_4_^+^ medium. **f**, **g** Dose-response relationship of the primary root elongation of Col-0 seedlings plotted as a function of the concentration of ROS scavengers. Data shown are mean ± SD. *n* = number of seedlings. *P* values < 0.05 indicate significant differences (one-way ANOVA with post-hoc Tukey HSD test). **h** Images of the effects of 750 µM dimethyl thiourea (DMTU) and 200 µM 4-hydroxy-TEMPO (TEMPO) on Col-0 seedlings. Four-day-old seedlings of the indicated genotypes were transferred to NO_3_^−^ or NH_4_^+^ medium with Fe^suff^ (100 µM) or Fe^low^ (10 µM) with or without the indicated ROS scavengers. Analyses were performed 4 days after seedling transfer. The experiments (**c**–**h**) were repeated independently three times with similar results, and representative data from one experiment are shown.

We subsequently focused on DEGs in the NH_4_^+^-N source. There were 182 DEGs (101 upregulated; 81 downregulated) between two Fe treatments for Col-0 seedlings and 130 DEGs (58 upregulated; 72 downregulated) between two genotypes that received Fe^suff^ treatment (Fig. 5a and Supplementary Fig. 19c, d). In these two comparisons, however, we did not find any DEGs currently known to be related to NH_4_^+^ toxicity (Supplementary Data 1f). Interestingly, the ratio of overlapped DEGs in the above two comparisons was high (Fig. 5a), and Gene Ontology (GO) enrichment analysis showed that the biggest cluster in both comparisons was related to “response to oxidative stress” (Fig. 5b).

The above results prompted us to monitor ROS formation in roots. We first histologically visualized ROS using 2’,7’-dichlorodihydrofluorescein diacetate (H_2_DCFDA), a fluorescent ROS indicator. The data showed that NH_4_^+^ resulted in clear ROS formation in root stele, which depended on either LPR2 action or sufficient Fe supplementation (Fig. 5c). The ratiometric fluorescent reporter protein roGFP2-Orp1 was recently shown to report the level of intracellular H_2_O_2_^29^. Because of the passage of extracellular H_2_O_2_ across the plasma membrane via aquaporins^30,31^, the extracellular H_2_O_2_ formation can also be indirectly reflected by roGFP2-Orp1 reporter in its neighboring cells^29^. Consistent with the observed ROS formation, the roGFP2-Orp1 reporter showed that NH_4_^+^ also triggered a burst of H_2_O_2_ in root stele under Fe^suff^ conditions (Supplementary Fig. 20). A close-up view of H_2_DCFDA-stained roots revealed that the NH_4_^+^-triggered burst of ROS mainly occurred in the phloem (Fig. 5d) and resembled the pattern of NH_4_^+^-induced Fe deposition. This finding was further supported by the roGFP2-Orp1 reporter in that the NH_4_^+^-induced H_2_O_2_ accumulation was overlaid well with the phloem marker esculin (Fig. 5e).

### ROS act downstream of Fe deposition to mediate growth response to NH_4_^+^

The above results implied that the remodeling of root development in response to NH_4_^+^ stress may be associated with a burst of ROS. Therefore, ROS scavengers were used to test this assumption. Unexpectedly, the addition of either ascorbic acid (ASA) or glutathione (GSH) to the NH_4_^+^ medium with Fe^suff^ resulted in greater inhibition of PR growth. This is probably because either ASA or GSH can enhance the production of hydroxyl radicals (·OH)—one of the most deleterious ROS—under Fe-rich conditions by Fenton reactions^32^. We also tested the effect of the H_2_O_2_ scavenger potassium iodide, but this compound showed high toxicity to plants in our growth condition because it could inhibit PR growth even in the NO_3_^-^ medium. Thus, we used other milder ROS scavengers, including dimethyl thiourea (DMTU) and 4-hydroxy-TEMPO (TEMPO), to counteract excess ROS production. Application of either of these scavengers to Col-0 seedlings had little effect on the growth of PRs in NO_3_^-^ medium with Fe^suff^ (Supplementary Fig. 21) but significantly restored PR growth in NH_4_^+^ medium with Fe^suff^ in a dose-dependent manner (Fig. 5f–h). The result suggests that Fe-dependent PR growth inhibition in NH_4_^+^-N source could be associated with excessive ROS formation.

We also examined Fe deposition after DMTU and TEMPO treatments. Although the PR growth of Col-0 seedlings in Fe^suff^ NH_4_^+^ medium was restored in the presence of ROS scavengers, Fe deposition in root phloem was still observed (Supplementary Fig. 22). Therefore, ROS very likely act downstream of Fe deposition to remodel root development in the NH_4_^+^-N source. However, application of either ROS scavenger lengthened the distance between the starting site of NH_4_^+^-induced Fe deposition and the root tip. This is probably associated with improved development of the root growth zone owing to ROS scavengers.

### NH_4_^+^ induces callose deposition in the phloem

**Fig. 6:**
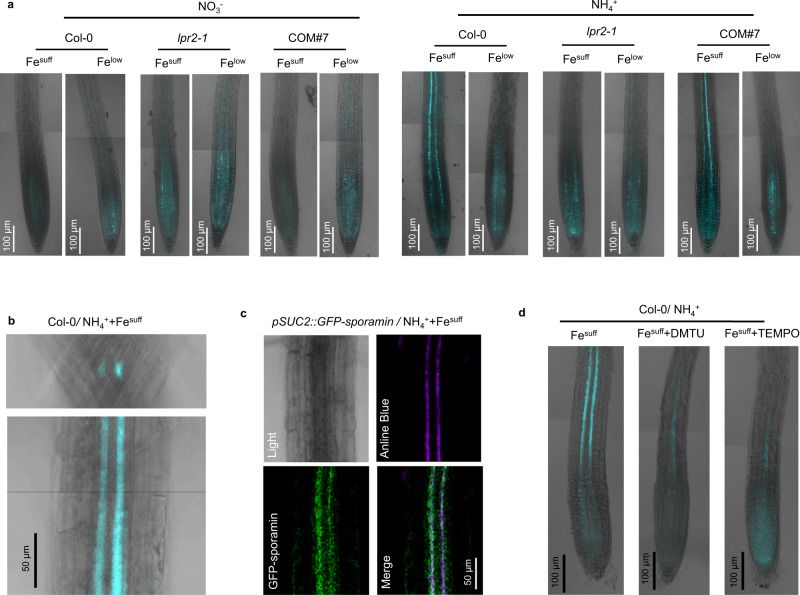
Fe-dependent callose deposition in the phloem via reactive oxygen species (ROS) in response to NH4+. **a** Callose detection in primary roots of Col-0, *lpr2-1*, and complementation line COM#7 seedlings by aniline blue staining. **b** Radial section (top) and close-up view (bottom) of aniline blue staining in primary roots of Col-0 seedlings grown in Fe^suff^ NH_4_^+^ medium. **c** Fluorescence co-localization of GFP-sporamin and aniline blue staining in *pSUC2::GFP-sporamin* roots. **d** ROS scavengers abolished the NH_4_^+^-induced callose deposition. Four-day-old seedlings of the indicated genotypes were transferred to NO_3_^−^ or NH_4_^+^ medium with Fe^suff^ (100 µM) or Fe^low^ (10 µM) with or without the indicated ROS scavenger. Each experiment was repeated independently three times with similar results, and a representative experiment is shown.

There is growing evidence that ROS regulates callose deposition^33^. We, therefore, examined callose formation by aniline blue staining (Fig. 6a). For Fe^suff^-treated Col-0 seedlings, the NH_4_^+^-N source resulted in massive callose deposition in root stele relative to that with the NO_3_^−^-N source. This NH_4_^+^-induced callose deposition was completely inhibited by either disruption of LPR2 in *lpr2-1* seedlings or Fe^low^ treatment in Col-0 seedlings. The complementation line COM#7 behaved similarly to Col-0. Therefore, NH_4_^+^ induces callose deposition in root stele in LPR2- and Fe-dependent manners. Furthermore, both the close-up and the radial section views of the Fe^suff^ NH_4_^+^ Col-0 roots showed that callose deposition was mainly localized in two poles of the root stele (Fig. 6b).

To determine the cell type for these two poles, aniline blue staining was performed in roots of *pSUC2::GFP-sporamin* transformants in which the GFP-sporamin marker was restricted in the phloem files, including sieve elements and companion cells^34^. Confocal analysis revealed that the aniline blue staining overlaid well with the GFP-sporamin marker in the roots subjected to Fe^suff^ NH_4_^+^ treatment, showing that NH_4_^+^-dependent callose deposition is localized in the phloem (Fig. 6c). At high magnification, callose was found to be located at the periphery of sieve elements (Supplementary Fig. 23), which is strikingly similar to the localization of the Fe deposition.

It is worth noting that, although neither DMTU nor TEMPO abolished the NH_4_^+^-induced Fe deposition in the root phloem (Supplementary Fig. 22), they substantially blocked callose deposition in the phloem (Fig. 6d). Therefore, the burst of ROS acts downstream of Fe deposition to induce callose deposition in response to NH_4_^+^ stress.

### Impaired phloem action is responsible for the growth response to NH_4_^+^

**Fig. 7:**
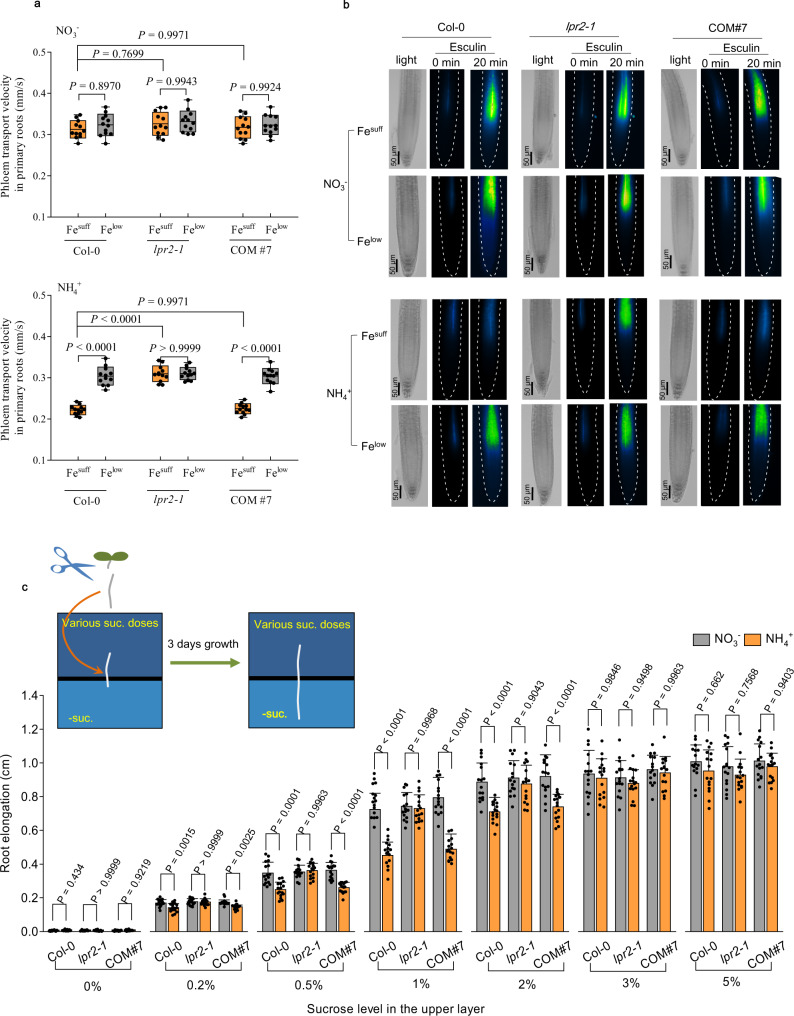
Inhibition of phloem action and sucrose complementation of primary root growth in NH4+ medium. **a**, **b** Phloem transport velocity and phloem unloading of esculin. Center line represents mean and bounds of box are SD; whiskers indicate the minimum and maximum values. *P* values < 0.05 indicate significant differences (two-way ANOVA with post-hoc Tukey HSD test, *n* = 12 seedlings per line and condition). Four-day-old seedlings of Col-0, *lpr2-1*, and complementation line COM#7 were transferred to NO_3_^−^ or NH_4_^+^ medium with Fe^suff^ (100 µM) or Fe^low^ (10 µM) and analyzed 4 days after seedling transfer. **c** Growth response of detached primary roots to localized sucrose supply. Data shown are mean ± SD. *P* values < 0.05 indicate significant differences (two-way ANOVA with post-hoc Tukey HSD test, *n* = 16 detached primary roots per line and condition). Inset: Scheme depicting the growth response analyses of detached primary roots to localized sucrose supply. Roots (1 cm) were cut from four-day-old seedlings. The detached roots were then transferred to a vertical two-layer split agar system of either NO_3_^−^ or NH_4_^+^ medium with Fe^suff^ or Fe^low^. The upper layers were treated with various doses of sucrose, and the lower layers were absent of sucrose. Analyses were performed 3 days after seedling transfer. Each experiment was repeated independently three times similar results, and a representative experiment is shown.

As callose deposition in the phloem may affect phloem action, the phloem-mobile probe esculin^35^ was used to evaluate the impacts of NH_4_^+^ stress on phloem action. The phloem transport velocity (PTV) was determined by dividing the distance of esculin traveled between two marks by the elapsed time^36^. The data showed that NH_4_^+^ significantly inhibited the PTV in PRs of Col-0 seedlings compared with the NO_3_^−^-N source under Fe^suff^ conditions, whereas either disruption of LPR2 in *lpr2-1* mutants or Fe^low^ treatment in Col-0 seedlings completely reversed this inhibition. The complementation line COM#7 behaved similarly to Col-0 (Fig. 7a). We then focused on phloem unloading. Once the esculin was imported into the root tip, individual images were recorded along with their times of acquisition^37^. As expected, NH_4_^+^ strongly arrested esculin unloading from phloem in the root growth zone in LPR2- and Fe-dependent manners (Fig. 7b). These results demonstrate that NH_4_^+^ impairs phloem function via the actions of LPR2 and Fe.

As esculin is loaded into the phloem by the sucrose transporter SUC2^35,38^, the impaired phloem transport and unloading of esculin reflect the inhibition of sucrose supply to the root growth zone. We, therefore, examined whether the LPR2-dependent NH_4_^+^-sensitive phenotype was associated with insufficient sucrose allocation to the root growth zone. Because all the above tests were performed using seedlings fed with 1% sucrose, we first needed to clarify an artificial effect from exogenous sucrose. The *lpr2-1* mutants still exhibited better PR growth than Col-0 seedlings did in the sucrose-free NH_4_^+^ medium, although the difference between them was slightly diminished compared with that in plants supplemented with 1% sucrose (Supplementary Fig. 24). Therefore, the genotype-specific growth response to the N source largely depends on the native biological processes of plants.

In subsequent tests, we used detached PRs to eliminate the potential effects of the sucrose source of shoots from different genotypes on their root growth. The detached PRs were transferred to a vertical two-layer split agar system with sucrose absent in the lower layer and various doses of sucrose present in the upper layer (Fig. 7c). In sucrose-free treatment, the detached PRs almost completely lost growth ability regardless of N source and genotype, indicating the importance of phloem-transported sucrose in maintaining root growth. For Col-0 seedlings, the growth difference of detached PRs between NO_3_^−^-N and NH_4_^+^-N sources with Fe^suff^ highly depended on the dose of sucrose: the difference was the most significant with 1% sucrose treatment, but it was diminished with lower sucrose doses because PR growth was inhibited in both N sources. Notably, the inhibitory effect of NH_4_^+^ on the growth of detached PRs of Col-0 seedlings was completely uncoupled by the elevation of sucrose to 3% or more. The detached PRs of *lpr2-1* mutants showed similar growth between the two N sources in all sucrose treatments, probably because LPR2 disruption protected the phloem from impairment due to NH_4_^+^ stress. The complementation line COM#7 behaved similarly to Col-0. Taken together, the above results point to a connection between the effect of NH_4_^+^-induced stress on root morphological response and insufficient sucrose allocation resulting from inhibited phloem action.

## Discussion

NH_4_^+^ can be directly assimilated after absorption into plant cells, whereas NO_3_^−^ needs to be reduced to NH_4_^+^ in an energy-consuming manner before it can be utilized by plants^39,40^. This raises a critically puzzling question because, although NH_4_^+^ is a theoretically preferred N source for plants, it inhibits PR growth when it is the only or predominant N source. Our study provides insights into the related factors that contribute to the adjustment of PR development in response to NH_4_^+^ stress by revealing the essential role of phloem Fe deposition in determining the NH_4_^+^-sensitive phenotype of PRs. Furthermore, increased external Fe availability intensifies NH_4_^+^-induced PR growth inhibition (Fig. 3a). Therefore, the occurrence of NH_4_^+^ toxicity in roots is not a single-factor event: Fe is also involved in this process. Notably, the conditions of soils that favor NH_4_^+^ formation also favor Fe solubilization. For example, in wetlands with anoxic conditions, the levels of soluble Fe can easily exceed 10 mg kg^−1^ (~180 µmol kg^−1^)^41,42^; in acidic soils rich in NH_4_^+^ due to higher ammonification than nitrification rates^43^, the low pH also greatly increases Fe solubility^44^. This close relationship between NH_4_^+^ and Fe in both plants and soils should be considered when developing agronomic strategies or genetically manipulated plants to cope with NH_4_^+^ stress.

The ferroxidase LPR2 is crucial for NH_4_^+^-induced Fe deposition in the phloem (Fig. 3c). Unexpectedly, the area of LPR2 distribution in root stele is much larger than the region of phloem-specific Fe deposition. Because LPR2 mainly resides in the cell wall matrix (Fig. 2a, b), the source of LPR2 substrate from a phloem cell-specific Fe^2+^ efflux may affect the specificity of NH_4_^+^-induced Fe deposition in the phloem. A comprehensive map of the microarray expression profiles of root cell types showed that *FPN1* is mainly expressed in phloem cells^45^. Therefore, the Fe^2+^ efflux protein FPN1 may also play a role in controlling the phloem specificity of Fe deposition. Nevertheless, disruption of FPN1 in T-DNA *fpn1* insertion mutants did not improve PR growth under NH_4_^+^ stress as it did in *lpr2* mutants (Supplementary Fig. 25), refuting the above-assumed role of FPN1. Another possible mechanism is that the allocation of photosynthesis products via phloem may allow the cell wall matrix of phloem cells and their adjacent cells to accumulate greater reductants, which favors producing Fe^2+^ for LPR2-catalyzed reactions. Regardless of the origin of Fe^2+^ in the cell wall matrix of phloem, the massive Fe deposition in the phloem due to NH_4_^+^ stress closely depends on the action of LPR2.

Interestingly, Fe is also a key player in remolding PR development in response to inorganic phosphate (_Pi_) deficiency, which mainly depends on the actions of LPR1^23,46–50^. However, although the amino acid sequences of LPR1 and LPR2 are 79% identical^23^, only LPR2 remolds PR development in the presence of NH_4_^+^ as the primary N source (Fig. 1). The failure to restore the NH_4_^+^-sensitive phenotype in *lpr2* mutants by confining *LPR1* expression to the LPR2 action site (Fig. 2e, f) suggests that the enzymatic actions of LPR1 and LPR2 in plants may be differently regulated. Furthermore, the tissue distribution of LPR1 also differs from that of LPR2 (Fig. 4 and Supplementary Fig. 15). The above differences may explain the specificity of LPR2 in mediating NH_4_^+^ sensitivity of PRs. Importantly, the *Km* value of recombinant GST-LPR2 (~17 µM Fe^2+^; Fig. 2d) is higher than that of LPR1 (~3 µM Fe^2+^)^27^. This discrepancy corresponds well with the discrepancy in Fe dose requirements in regulating PR growth responses to _Pi_ deficiency and NH_4_^+^ stress: 2.5 µM Fe was sufficient for LPR1 to inhibit PR growth under _Pi_ deficiency^27^, but an Fe dose of more than 10 µM was required for LPR2 to inhibit PR growth under NH_4_^+^ stress (Fig. 3).

Fe is a co-factor for several ROS-producing enzymes, such as NADPH oxidases, cytochrome P450 enzymes, lipoxygenases, and xanthine oxidase^51^. Fe redox cycling is also capable of generating ROS directly via Fenton reactions^52^. Therefore, aberrant accumulation of Fe often triggers direct and indirect bursts in ROS^53^. Regardless of the means of Fe regulation of ROS generation, our study showed a clear burst in ROS in root phloem as a result of NH_4_^+^-induced Fe deposition (Fig. 5c–e). However, we were unable to distinguish whether this burst was generated in the intra- or extracellular matrix of phloem. Considering the apoplastic Fe deposition in the phloem, the NH_4_^+^-induced burst of ROS is most likely an extracellular process. Studies have revealed a strong connection between callose deposition and bursts of ROS in response to external biotic and abiotic cues^33^. The ROS burst is also responsible for the induction of callose deposition in response to NH_4_^+^ stress (Fig. 6d). The _Pi_ deficiency-induced PR growth inhibition was identified to be associated with enhanced callose accumulations in the elongation zone and SCN, which mainly depends on LPR1-mediated Fe deposition^26,47^. Unexpectedly, our study showed that although Fe deposition was also observed in the SCN in all treatments, particularly under Fe^low^ conditions, the callose in this region did not accumulate correspondingly across the treatments (Figs. 3c and 6a). Callose is degraded by β-1,3-glucanases^54^. Bioinformatic analyses have identified approximately 50 β-1,3-glucanase-related genes in Arabidopsis^55^. A recent single-cell RNA-Seq showed that several β-1,3-glucanase-related genes, such as *At1g66250*, *At2g05790*, *At2g01630* (*PdBG2*), *At4g29360*, *At5g42100*, and *At5g58090*, are preferentially expressed in root SCN^56^. The impacts of these β-1,3-glucanases in callose accumulation in the SCN may vary in different treatments.

The phloem is implicated as an important mediator of plant growth plasticity in response to environmental cues^57^. Under normal growth conditions, callose is present in the sieve plates and plasmodesmata neck region of phloem at a basal level. Heavy deposition of callose in the phloem may plug sieve pores and decrease plasmodesmal permeability^54,58^. Indeed, our phloem-mobile probe test using esculin provides direct evidence that NH_4_^+^-induced callose deposition significantly inhibits phloem transport and unloading. Sucrose is the major transport sugar in the phloem from cotyledon or mature leaves to roots, while root growth depends highly on the sucrose imported from shoot parts^57^. Esculin is loaded into the phloem in a strict sucrose transporter-dependent manner^35,38^. Therefore, the NH_4_^+^-induced inhibition of phloem action, as indicated by esculin (Fig. 7a, b), suggests that the sucrose supply to the root growth zone may be insufficient for sustaining normal cell division and differentiation. This idea is supported by the finding that elevation of sucrose supplementation in the upper part of detached roots completely rescued PR growth under NH_4_^+^ stress (Fig. 7c). Previously, several physiological issues, including depletion of the carbon supply, deficiency of mineral cations (i.e., K^+^), excess root energy demands associated with NH_4_^+^ assimilation, and high energetic cost due to futile transmembrane NH_4_^+^ cycling, were proposed as toxic actions of NH_4_^+^^1,59^. However, these actions may be related to the inhibition of sucrose transport in the phloem due to aberrant Fe deposition. This is because insufficient sucrose supply would not only directly lead to carbon depletion but also result in failure to meet the energy demands for either excess NH_4_^+^ assimilation or futile transmembrane NH_4_^+^ cycling. In addition, the sucrose loading into phloem requires the action of K^+^ loading^60^, and thus a deficiency of K^+^ caused by NH_4_^+^ stress could further result in inhibition of sucrose transport in the phloem. Within these contexts, inhibition of phloem action as a consequence of Fe deposition could be a primary mechanism that connects the inhibition of root development to NH_4_^+^ stress.

It is worth noting that, in addition to transporting sugar, phloem also delivers other numerous substances, including proteins, RNAs, and peptides, but currently, only a few have been confirmed to be associated with a function^61^. Recently, the movement of 5-methylcytosine-modified AtTCTP1 mRNA in the phloem was shown to slightly promote PR growth of Arabidopsis^62^. In addition, the protophloem has been proposed as a nexus that senses CLE peptides that convey environmental conditions and adjusts root growth accordingly^63^. In these contexts, the action of phloem in regulating PR growth is not merely limited to the movement of sucrose. Therefore, the roles of other substances, particularly signal molecules, in the regulation of PR growth inhibition from a resulting impairment of phloem action under NH_4_^+^ stress might merit further study.

**Fig. 8:**
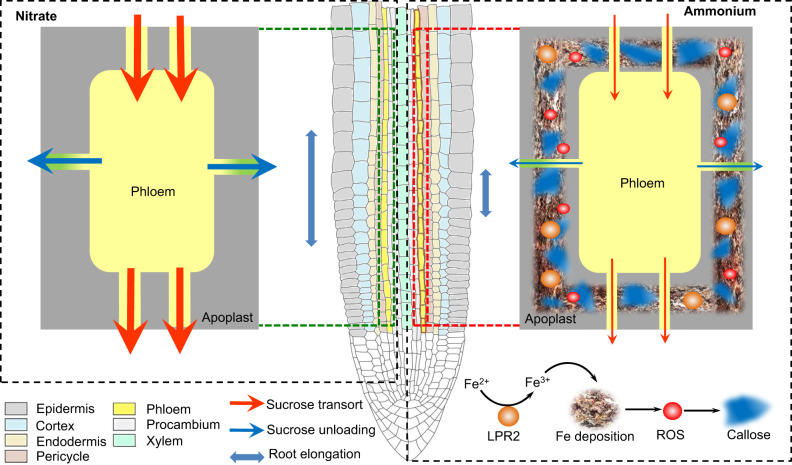
Schematic model of the mechanism that connects remodeling of root development to NH4+ as the primary N source. In comparison with NO_3_^−^ as the primary N source, NH_4_^+^ supplementation as the predominant N source induces excessive Fe deposits in the apoplast of root phloem via a catalytic reaction of Fe^2+^ oxidation to Fe^3+^, which depends on the action of the cell wall-localized ferroxidase LPR2 in root stele. Such an aberrant Fe accumulation then inhibits phloem transport and unloading by triggering a burst of reactive oxygen species, which subsequently induces massive callose deposition. Consequently, sucrose is insufficiently allocated to the root growth zone, which arrests root development.

In conclusion, we propose a mechanism by which root development is remolded in response to a variation in the predominant N source (Fig. 8). In comparison with NO_3_^−^ as the major N source, NH_4_^+^ induces excessive Fe deposits in the apoplast of root phloem via the action of cell wall-localized ferroxidase LPR2, which catalyzes the oxidation of Fe^2+^ into Fe^3+^. Such an aberrant Fe accumulation triggers ROS generation and consequently leads to massive callose deposition. This impairs phloem action and arrests root development because of insufficient sucrose allocation to the root growth zone. Our findings may provide a strategy for improving plant adaptation to NH_4_^+^-rich soil environments via biotechnological pathways to manipulate Fe deposition in root phloem.

## Methods

### Plant lines and growth conditions

Wild-type *A. thaliana* accession Columbia (Col-0) was used as a control for all experiments. The T-DNA insertion lines *lpr2-1* (SALK_091930), *isas* (SALK_056696), and *fpn1* (SALK_055499) were obtained from the Arabidopsis Biological Resource Center (Ohio State University, Columbus, OH, USA). The *lpr1-1* and *lpr1-1lpr2-1* (*lpr1lpr2*) mutants were provided by Prof Liu Dong (Tsinghua University, Beijing, China); *pSUC2::GFP-sporamin* and roGFP2-orp1 were provided by Prof Chun-Ming Liu (Chinese Academy of Sciences, Beijing, China) and Dr Markus Schwarzländer (University of Münster, Münster, Germany), respectively. Homozygous lines were used in this study, and the insertions were verified using the primers listed in Supplementary Data 2.

The plants were grown on a basic agar medium in sterile Petri dishes. Briefly, the seeds were surface-sterilized using 25% NaClO and sown on basal medium containing 1% (w/v) sucrose, 0.8% (w/v) agar, and MES. The nutrient composition of the basal medium was as follows: 3 mM KNO_3_, 0.5 mM (NH_4_)_2_SO_4_, 1 mM CaCl_2_, 500 μM NaH_2_PO_4_, 500 μM MgSO_4_, 100 μM Fe(II)-EDTA, 10 μM H_3_BO_3_, 0.5 μM ZnSO_4_, 0.5 μM MnSO_4_, 0.1 μM (NH_4_)_6_Mo_7_O_24_, and 0.1 μM CuSO_4_ (pH 5.7). After vernalization at 4 °C for 2 days, the Petri dishes were moved to a controlled-environment growth chamber with a 12 h light/12 h dark cycle at 22 °C. The 4-day-old seedlings were then transferred to basic agar media with 2.5 mM (NH_4_)_2_SO_4_ or 5 mM KNO_3_ as the sole N source, containing various doses of Fe(II)-EDTA as indicated in the figure legends. The resulting differences in K concentrations were balanced by adjusting the KCl concentration. To avoid a possible artificial impact from light irradiation on roots, the roots of seedlings were covered with a sterile black plastic sheet, as depicted in Supplementary Fig. 3a.

### Generation of transgenic plants

For the generation of plant expression vectors for genetic complementation, the 5949-bp genomic DNA of *LPR2* (including a 2928-bp region upstream of the start codon) and the 4262-bp genomic DNA of *LPR1* (including a 2178-bp region upstream of the start codon) were cloned into a pEarleyGate 101 vector containing a YFP reporter. The resulting constructs *pLPR2::LPR2-YFP* and *pLPR1::LPR1-YFP* were then transformed into *lpr2-1* and *lpr1-1* mutants, respectively, to generate complementation lines (*pLPR2::LPR2-YFP*/*lpr2-1* and *pLPR1::LPR1-YFP*/*lpr1-1*, respectively). For analysis of subcellular localization of LPR2, the *LPR2* coding sequence (CDS), fused to GFP, was cloned into a modified pCAMBIA1300 vector containing a doubled CaMV 35S promoter. The resulting construct was transformed into Col-0 to obtain the *p35S::LPR2-GFP* line. For the *LPR2* promoter-confined complementation assay of *LPR1*, the 2928-bp region upstream of the start codon of *LPR2*, together with either the CDS or genomic sequence of *LPR1*, was cloned into a pCAMBIA1300 vector. The resulting construct was transformed into *lpr2-1* mutants to generate *pLPR2::LPR1*^*CDS*^/*lpr2-1* or *pLPR2::LPR1*^*genomic*^/*lpr2-1* transformants.

All primers used for vector construction are listed in Supplementary Data 2. All transgenic lines were generated by agrobacterium-mediated transformation using the floral dip method. Only homozygous lines were used in this study.

### Measurements of root meristem size and cell elongation

Measurements of root meristem size and cell elongation were performed 4 days after seedling transfer to the indicated media. The roots were incubated in 10 μg mL^–1^ propidium iodide (PI; Sigma-Aldrich, St. Louis, MO, USA) in the dark for 5 min and rinsed twice with deionized water. The roots were then imaged by confocal microscopy. Root meristem size was measured as the number of cortical cells between the quiescent center and the first elongated cell (Supplementary Fig. 3b). The average number of elongating cells in the same cell file was calculated from the first elongating cell to the first differentiating cell. The length of the latter cell type was measured to calculate the average length of the first differentiating cells.

### Histochemical Fe staining

The histochemical Fe staining of roots was adapted from the method of Roschzttardtz et al.^22^. Briefly, after 4 days of treatments in the indicated media, the roots were incubated for 45 min under vacuum (500 mbar) with Perls’ staining solution (2% [v/v] HCl and 2% [w/v] potassium ferrocyanide). After being rinsed three times with deionized water, the root samples were incubated for 1 h in a methanol solution containing 10 mM NaN_3_ and 0.3% H_2_O_2_. Subsequently, the root samples were washed with 100 mM Na-phosphate buffer (pH 7.4) and intensified by 5 min incubation in the same buffer containing 0.025% (w/v) DAB and 0.005% (v/v) H_2_O_2_. The reaction was stopped by washing with deionized water and optically cleared with chloral hydrate. Stained root samples were observed using a differential interference contrast microscope (Nikon, Tokyo, Japan).

### Confocal microscopy and staining procedures

Confocal analyses were performed on either an Olympus FV3000 (Tokyo, Japan) or a Zeiss LSM 880 microscope (Oberkochen, Germany). For callose staining, roots were incubated in 150 mM K_2_HPO_4_ and 0.01% (w/v) aniline blue in the dark for 2 h. For ROS staining, roots were incubated with 10 μM H_2_DCFDA (Beyotime Biotech, Shanghai, China) in the dark for 30 min and then washed twice with deionized water. For PI or FM4-64 staining, roots were directly imaged in 10 μM PI or 4 μM FM4-64 (Thermo Fisher Scientific, Waltham, MA, USA). The excitation (ex)/emission (em) parameters for confocal analyses are as follows: GFP/YFP/H_2_CDFA ex: 488 nm, em: 500–550 nm; PI/FM4-64 ex: 561, em: 570–670 nm; aniline blue ex: 405 nm, em: 475–525 nm; esculin ex: 405 nm, em: 420–480 nm; and roGFP2-Orp1 ex: 405 and 488 nm in sequence, em: 505–535 nm and 425–475 nm (auto fluorescence recorded at ex: 405 nm for subtraction in calculating the ratio 405/488 nm)^29^.

### Analysis of esculin movement

The esculin movement in the phloem of PRs was detected as described by Knox^36^. Briefly, after 4-day treatment with the indicated media, cotyledons were pretreated with 0.3 μL 2.5% (v/v) Adigor solution (Syngenta, Basel, Switzerland) for 1 h and subsequently supplemented with 0.5 μL 9 mg mL^–1^ esculin solution. After 10 min, the appearance of esculin in the phloem of the PR was noted. Then, the fluorescent front in root phloem was marked on the plate, and the time was noted. Thereafter, the fluorescence in root phloem was re-checked after 10 min, and the new front was marked along with the time. The distance of esculin movement in the phloem was taken as the length between the two marks. The PTV was calculated from the distance and the interval time. In addition, once esculin was imported into the root tip, individual images, with their times of acquisition, were recorded to evaluate the unloading of the phloem unloading^37^.

### In vitro ferroxidase characterization of recombinant proteins

The *LPR2* CDS without signal peptide sequences were cloned into pGEX-6P-1, which enables the fusion of a GST tag. Isopropyl β-D-1 thiogalactopyranoside (0.5 mM) was supplied to *Escherichia coli* strain Arctic Express carrying pGEX-6P-1 or pGEX-6P-1-LPR2 at 14 °C for 20 h to induce expression of the GST or GST-LPR2. The cells were harvested by centrifugation (4000 × *g*, 30 min, 4 °C) and lysed in extraction buffer (50 mM Tris-HCl (pH 7.4), 150 mM NaCl, 1 mM PMSF, and 100 μg·mL^−1^ mL lysozyme) with sonication. After centrifugation at 15,000 × *g* at 4 °C for 30 min, the supernatant was recovered, then the GST or GST-LPR2 protein was purified using a column containing Glutathione Sepharose 4B beads (Solarbio, Beijing, China)^64^. The purified proteins were further desalted and concentrated using centrifugal filter (Merck Millipore, Amicon Ultra 15, molecular weight cut-off of 10 kDa).

Ferroxidase activity was measured using a ferrozine-based method^26^. Briefly, a defined amount of protein was mixed with various amounts of ferrous ammonium sulfate in a solution of 0.1 M sodium acetate with 100 μM CuSO_4_ (pH 5.0) to reach desired substrate concentrations. After incubation, 200-μL samples were taken in appropriate intervals and transferred to 96-well plates for reaction-quenching with 14 μL 18 mM ferrozine. The absorbance at 560 nm was measured using a plate reader (SpectraMax i3x; Molecular Devices, San Jose, CA, USA). The decrease in the substrate was then used to calculate the oxidation of Fe^2+^. The K_m_ and V_max_ were calculated using the Michaelis–Monod model in GraphPad Prism 8 software (GraphPad Software, San Diego, CA, USA).

### Determination of ammonium

Root samples were ground in liquid N_2_ immediately after collection. One milliliter of 0.3 mM sulfuric acid solution was added to the frozen powder, followed by vortexing. The mixture was centrifuged at 12,000 × *g* at 4 °C for 10 min. The NH_4_^+^ concentration in the supernatant was determined colorimetrically using a method based on the phenol-hypochlorite assay at 635 nm in a spectrophotometer^10^.

### Quantitative real-time RT–PCR

After 4-day treatment in the indicated media, the total RNA in roots was extracted using RNAisoPlus (Takara, Kyoto, Japan) and treated with DNase I to remove contaminant genomic DNA. We synthesized cDNA using a PrimeScript RT reagent kit (Takara) according to the manufacturer’s protocol. We performed qRT-PCR analysis of *LPR2* with TB Green Premix Ex Taq II (Takara) on a Step One system (Agilent Technologies, Santa Clara, CA, USA). The primers used for qRT-PCR analysis are listed in Supplementary Data 2. *UBQ10* and *EF1α* were used as the reference genes. Relative transcript abundances were determined by normalizing to the geometric mean of expression of two reference genes for each sample^65^.

### Immunoblot analysis

Polyclonal LPR2 epitope-specific antibody was raised in rabbits against a synthetic peptide (LPR2:DEGGIKQEERLFNLGKLE-C) and affinity-purified (ABclonal, Wuhan, China). After 4-day treatment in the indicated media, the total root proteins were extracted using a Plant Protein Extraction Kit (Solarbio, Beijing, China) following the manufacturer’s protocol. The protein samples were separated in 10% SDS/PAGE gels and transferred to nitrocellulose membranes (Bio-Rad, Hercules, CA, USA) in semi-dry conditions (Trans-Blot SD semi-dry transfer cell; Bio-Rad). The membranes were incubated in blocking buffer (TBST, 5% milk powder) at room temperature for 2 h and then incubated overnight in blocking buffer containing anti-LPR2 antibody (1:2000), rabbit polyclonal anti-GFP (1:5000; catalogue no. AE011, ABclonal, Wuhan, China) or mouse monoclonal anti-actin antibody (1:2000; catalogue no. D191048, Sangon Biotech, Shanghai, China) at 4 °C. HRP-conjugated goat anti-rabbit IgG (1:5000; catalogue no. D110058, Sangon Biotech) or goat anti-mouse IgG (1:5000, catalogue no. D110087, Sangon Biotech) was used as a secondary antibody. The signal was detected using an ECL Kit (Tanon™; AbClon). In addition, the membranes were also stained with Ponceau S to visualize total proteins as the loading reference.

### RNA sequencing and GO analysis

After 4-day treatment in the indicated media, the total RNA was extracted from the roots of Col-0 and *lpr2-1* seedlings using MagZol Reagent (Magen, Guangzhou, China) following the manufacturer’s protocol. We constructed mRNA-Seq libraries using NEBNext Ultra RNA Library Prep Kit for Illumina (NEB, Ipswich, MA, USA). The quality of the libraries was assessed using an Agilent 2100 Bioanalyzer. Sequencing was performed using a Hiseq Xten sequencer (Illumina, San Diego, CA, USA) at RIBOBIO (Guangzhou, China). Raw reads were quality controlled and trimmed using FastQC v0.11.8 (http://www.bioinformatics.babraham.ac.uk/projects/fastqc/) and Trimmomatic. The clean reads were mapped to the Arabidopsis genome (TAIR10) using HISAT2 and a GTF annotation file from the Ensembl Plants database (ftp://ftp.ensemblgenomes.org) and then analyzed using *Stringtie* and *ballgown*^66^. Significant DEGs were assessed using a *P* value threshold of <0.05 and |log_2_(fold change)| > 1 using DEGseq^67^. GO term enrichment analysis was performed with R packages clusterProfiler and database org.At.tair.db (version 3.13.0)^68^.

### Statistical analyses

Data were analyzed by one-way and two-way ANOVA with post-hoc Tukey HSD test using Prism v8 (GraphPad). A *P* value < 0.05 was considered statistically significant.

### Reporting summary

Further information on research design is available in the Nature Research Reporting Summary linked to this article.

## Supplementary information

Supplementary Information

Peer Review File

Description of Additional Supplementary Files

Supplementary Data 1

Supplementary Data 2

Reporting Summary

## Data Availability

Data supporting the findings of this work are available within the paper and its Supplementary Information files. A reporting summary for this article is available as a Supplementary Information file. RNA-seq raw data were deposited in NCBI’s SRA (Sequence Read Archive) under the accession number PRJNA789742. Source Data are provided with this paper.

## Source data

Source Data
