# Supplementary material for: Phloem iron remodels root development in response to ammonium as the major nitrogen source
Source: Nat Commun. 2022 Jan 28;13:561. doi: 10.1038/s41467-022-28261-4 (PMC8799741; doi:10.1038/s41467-022-28261-4)
Supplement: Supplementary file 1 — Supplementary Information [file 41467_2022_28261_MOESM1_ESM.pdf]

## Supplementary Figures

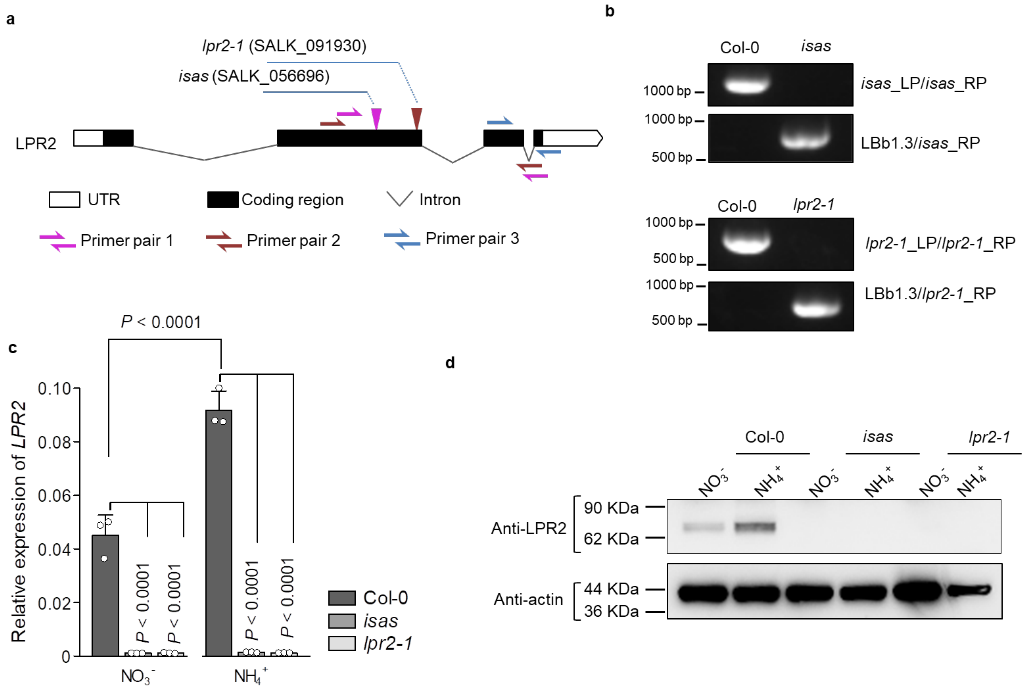


**Supplementary Figure 1 Characterization of *lpr2* mutants. a** Positions of T-DNA insertion in the *lpr2* mutants (*isas* and *lpr2-1*) and locations of primers used for genotyping and qRT-PCR. Primer pairs 1 and 2 were used for *isas* and *lpr2-1* genotyping, respectively. Primer pair 3 was used for qRT-PCR. **b** PCR-based genotyping. **c** *LPR2* expression in roots. Data shown are mean ± SD of three biological replicates. *P*-values < 0.05 indicate significant differences (two-way ANOVA with post-hoc Tukey HSD test). **d** Immunoblot of LPR2 in roots. Four-day-old seedlings were transferred to medium with $NO_3^-$ or $NH_4^+$ as the nitrogen source and 100 μM Fe supplementation and analyzed 4 d after seedling transfer. The experiment was repeated independently twice with similar results, and a representative experiment is shown.

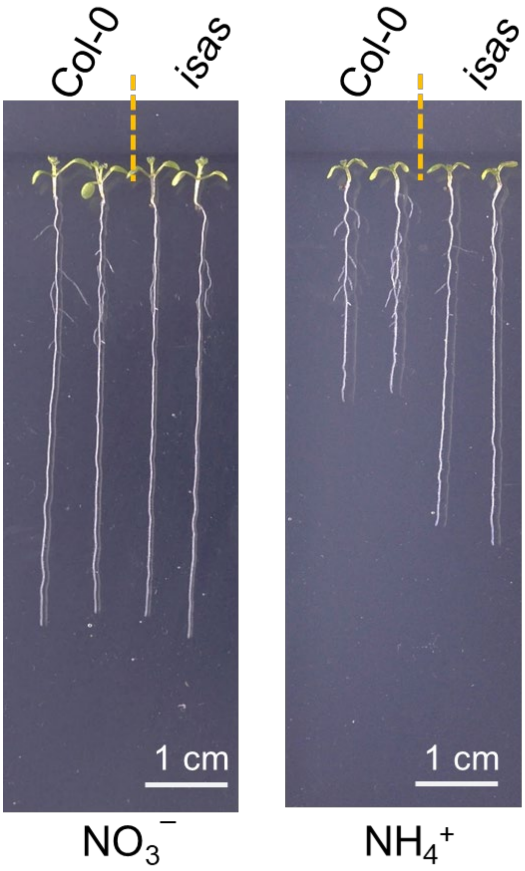


**Supplementary Figure 2 Growth response of primary roots of Col-0 and *isas* seedlings in light-irradiated $NH_4^+$ medium.** Four-day-old seedlings were transferred to light-irradiated rooting medium with $NO_3^-$ or $NH_4^+$ as the nitrogen source and 100 μM Fe supplementation and analyzed 4 d after seedling transfer. The experiment was repeated independently at least three times with similar results, and a representative experiment is shown.

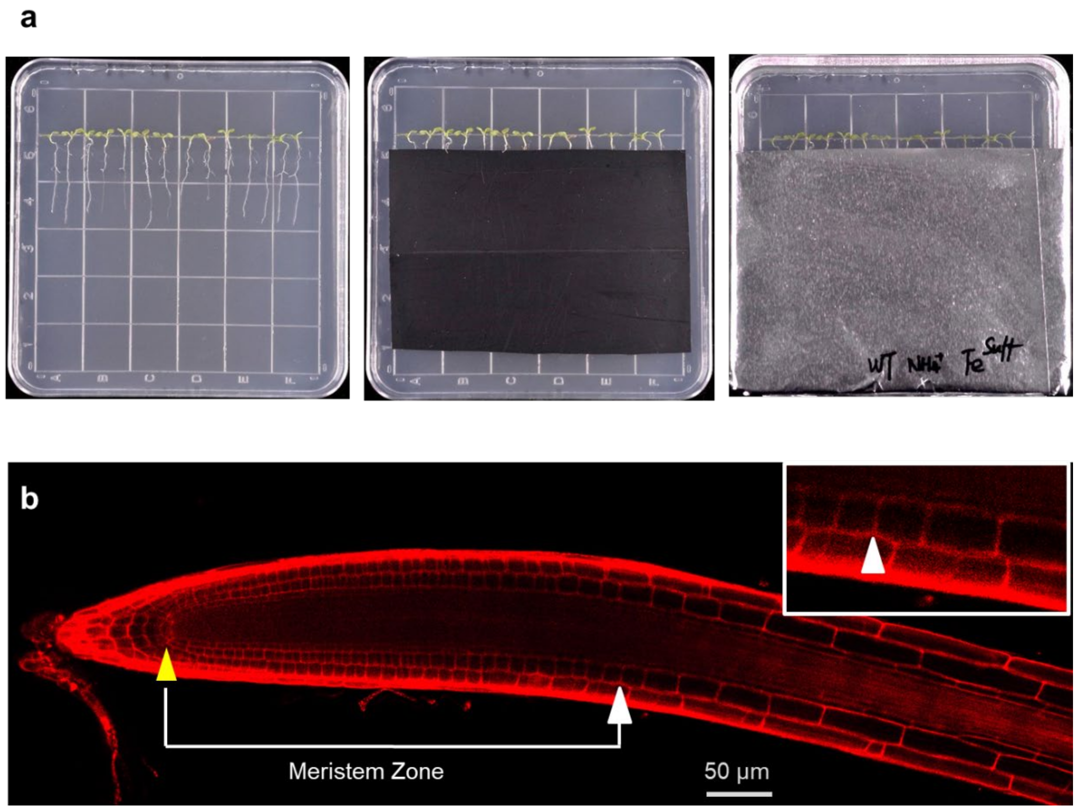


**Supplementary Figure 3 Photographs illustrating the methods for growing root-shaded seedlings and measuring root meristem size. a** Method for growing the root-shaded seedlings. The root exudates dissolved $Al^{3+}$ from the aluminum foil, which may be toxic to the roots; hence, we used a black plastic sheet to cover the rooting medium. Briefly, the roots of four-day-old seedlings were covered with a sterilized black plastic sheet while the shoots remained exposed to light. The periphery of the Petri dish below the shoots was covered with aluminum foil. **b** Method for measuring the meristem size of roots. The root meristem size was measured as the number of cortical cells between the quiescent center (yellow arrowhead) and the first elongated cell (white arrowheads). The roots were incubated in 10 µg $mL^{-1}$ propidium iodide for 5 min and rinsed with deionized water. The roots were then imaged by confocal microscopy. The experiment was repeated independently at least three times with similar results, and a representative experiment is shown.

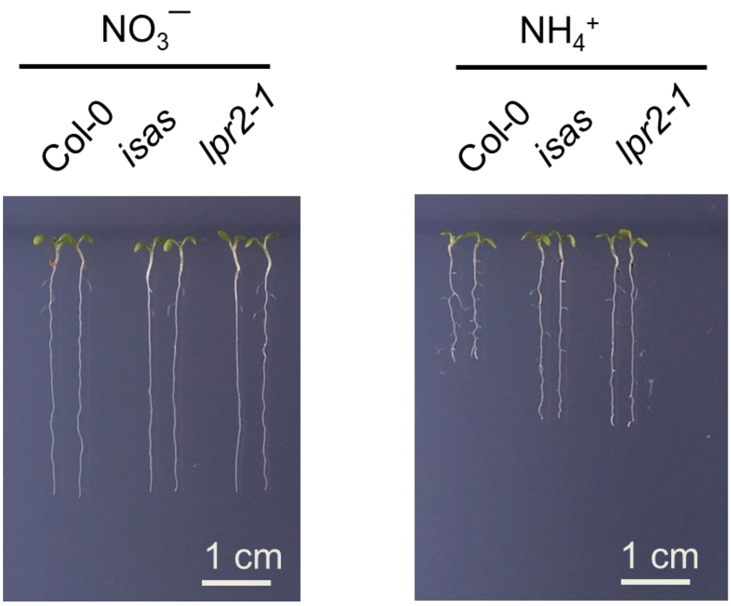


**Supplementary Figure 4 LPR2-dependent primary root growth inhibition in $NH_4^+$ medium without pH buffer MES**. Four-day-old seedlings of the indicated genotype were transferred to $NO_3^-$ or $NH_4^+$ medium with 100 μM Fe and without addition of pH buffer MES. The phenotype was analyzed 4 d after seedling transfer. The experiment was repeated independently twice with similar results, and a representative experiment is shown.

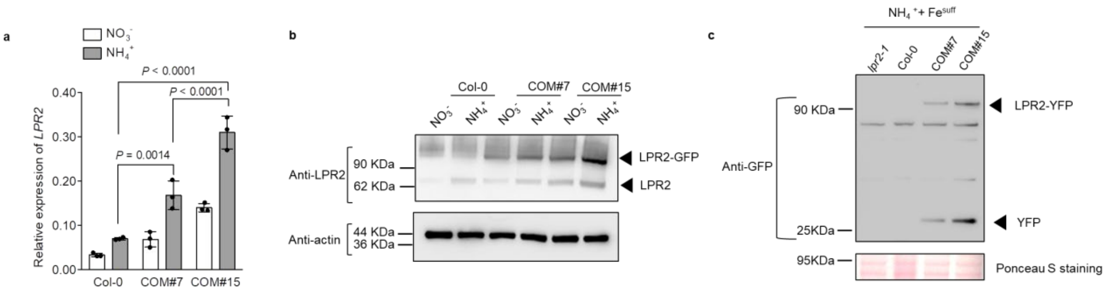


**Supplementary Figure 5 Characterization of *pLPR2::LPR2-YFP/lpr2-1* complementation lines. a** *LPR2* expression in roots. Data shown are mean ± SD of three biological replicates. *P*-values < 0.05 indicate significant differences (two-way ANOVA with post-hoc Tukey HSD test). **b** Immunoblot of LPR2 in roots. **c** Immunoblot of YFP in roots. The signals of untagged LPR2 and free YFP in two *pLPR2::LPR2-YFP/lpr2-1* complementation lines suggest a cleavage of the LPR2-YFP protein. Four-day-old seedlings of the indicated genotypes were transferred to medium with $NO_3^-$ or $NH_4^+$ as the nitrogen source and 100 μM Fe supplementation and analyzed 4 d after seedling transfer. Each experiment was repeated independently twice with similar results, and a representative experiment is shown.

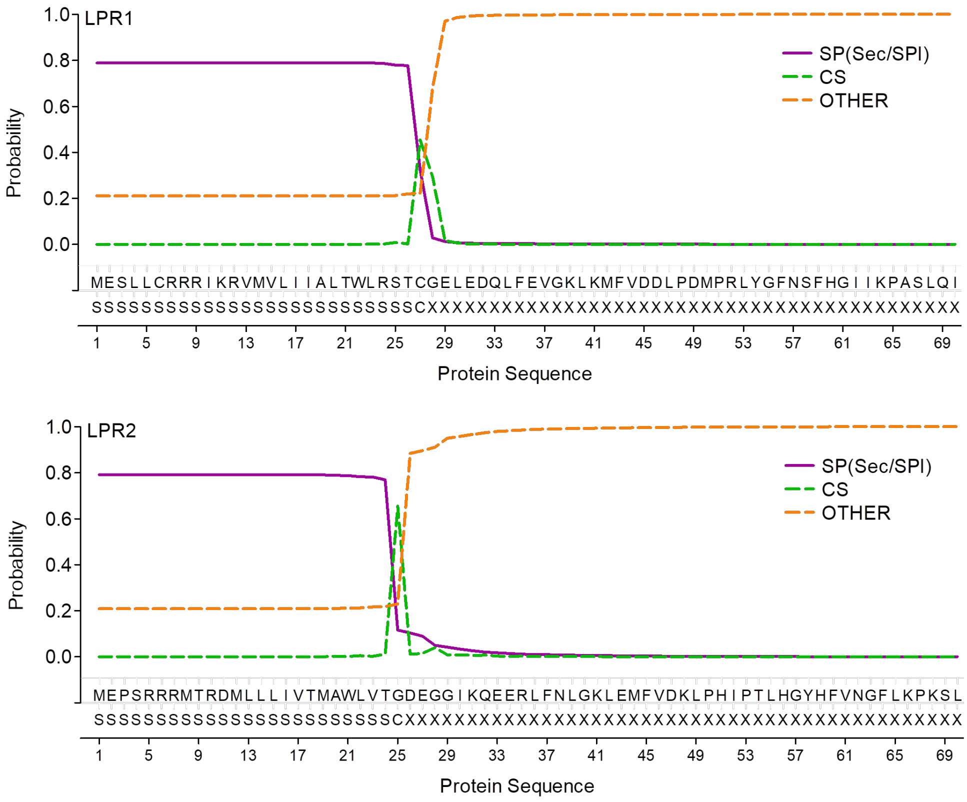


**Supplementary Figure 6 Predictions of signal peptides in LPR1 and LPR2.** The SignalP 5.0 algorithm (http://www.cbs.dtu.dk/services/SignalP/) was used to predict signal peptides. SP (Sec/SPI): type of signal peptide; CS: the cleavage site; OTHER: the probability that the sequence does not have any kind of signal peptide. Sec/SPI indicates secretory signal peptide transported by the secretion translocon and cleaved by signal peptidase I.

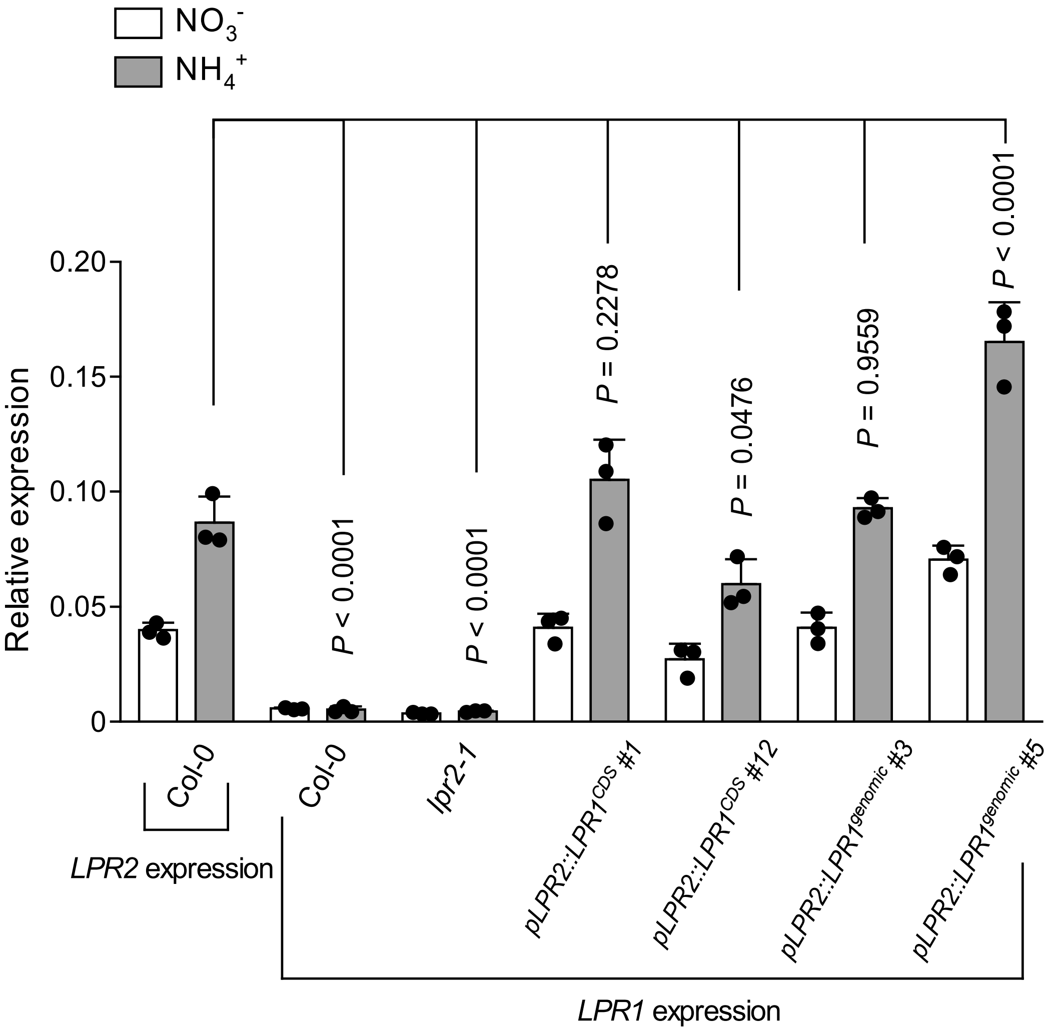


**Supplementary Figure 7 Comparison of the expression levels of *LPR1* in *pLPR2::LPR1/lpr2-1* lines and *LPR2* in Col-0 plants.** Four-day-old seedlings of the indicated genotype were transferred to medium with $NO_3^-$ or $NH_4^+$ as the nitrogen source and 100 μM Fe supplementation and analyzed 4 d after seedling transfer. Data shown are mean ± SD of three biological replicates. *P*-values < 0.05 indicate significant differences (one-way ANOVA with post-hoc Tukey HSD test). The experiment was repeated independently at twice with similar results, and a representative experiment is shown.

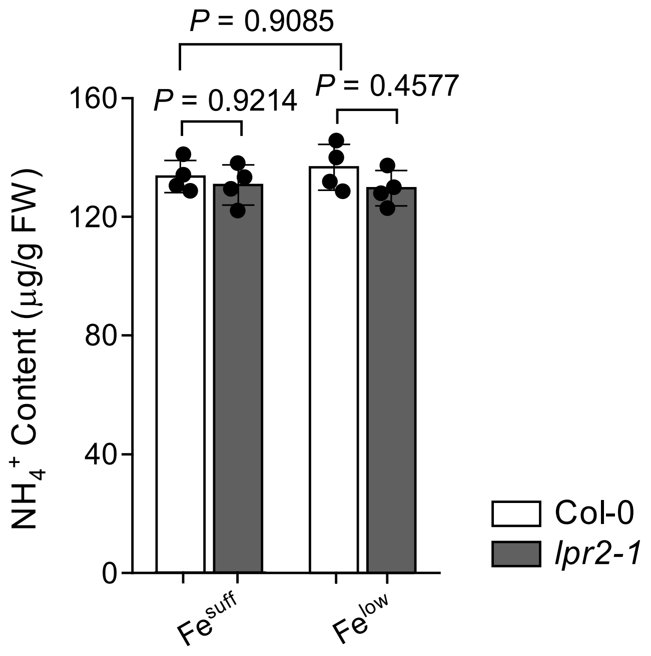


**Supplementary Figure 8 Root $NH_4^+$ concentrations in *lpr2-1* and Col-0 seedlings.** Four-day-old seedlings of the indicated genotype were transferred to $NO_3^-$ or $NH_4^+$ medium with $Fe^{suff}$ (100 µM) or $Fe^{low}$ (10 µM) and analyzed 4 d after seedling transfer. Data shown are mean ± SD of four biological replicates. *P*-values > 0.05 indicate nonsignificant differences (two-way ANOVA with post-hoc Tukey HSD test). Each experiment was repeated independently twice with similar results, and a representative experiment is shown.

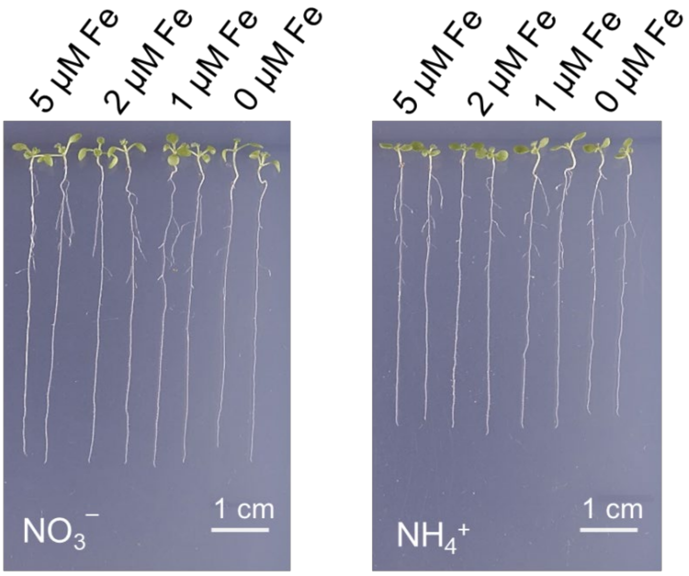


**Supplementary Figure 9 Growth response of primary roots of Col-0 seedlings in $NO_3^-$ or $NH_4^+$ medium with various low doses of Fe.** Four-day-old Col-0 seedlings of the indicated genotype were transferred to $NO_3^-$ or $NH_4^+$ medium with various doses of Fe supply and analyzed 4 d after seedling transfer. The experiment was repeated independently twice with similar results, and a representative experiment is shown.

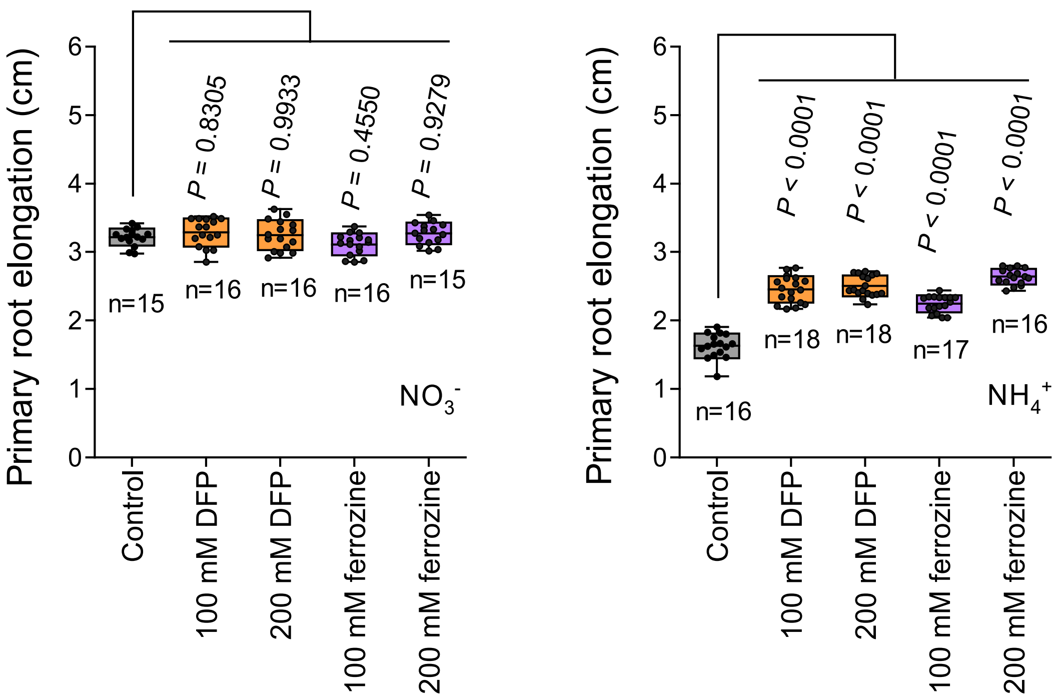


**Supplementary Figure 10 Effects of Fe chelators on the primary root growth of Col-0 seedlings.** Four-day-old Col-0 seedlings were transferred to $NO_3^-$ or $NH_4^+$ medium with $Fe^{suff}$ and with or without the indicated Fe chelators. Seedlings were analyzed 4 d after transfer. Centre line represents mean and bounds of box are SD; whiskers indicate the minimum and maximum values; n = number of seedlings. *P*-values < 0.05 indicate significant differences (one-way ANOVA with post-hoc Tukey HSD test). The experiment was repeated independently three times with similar results, and a representative experiment is shown.

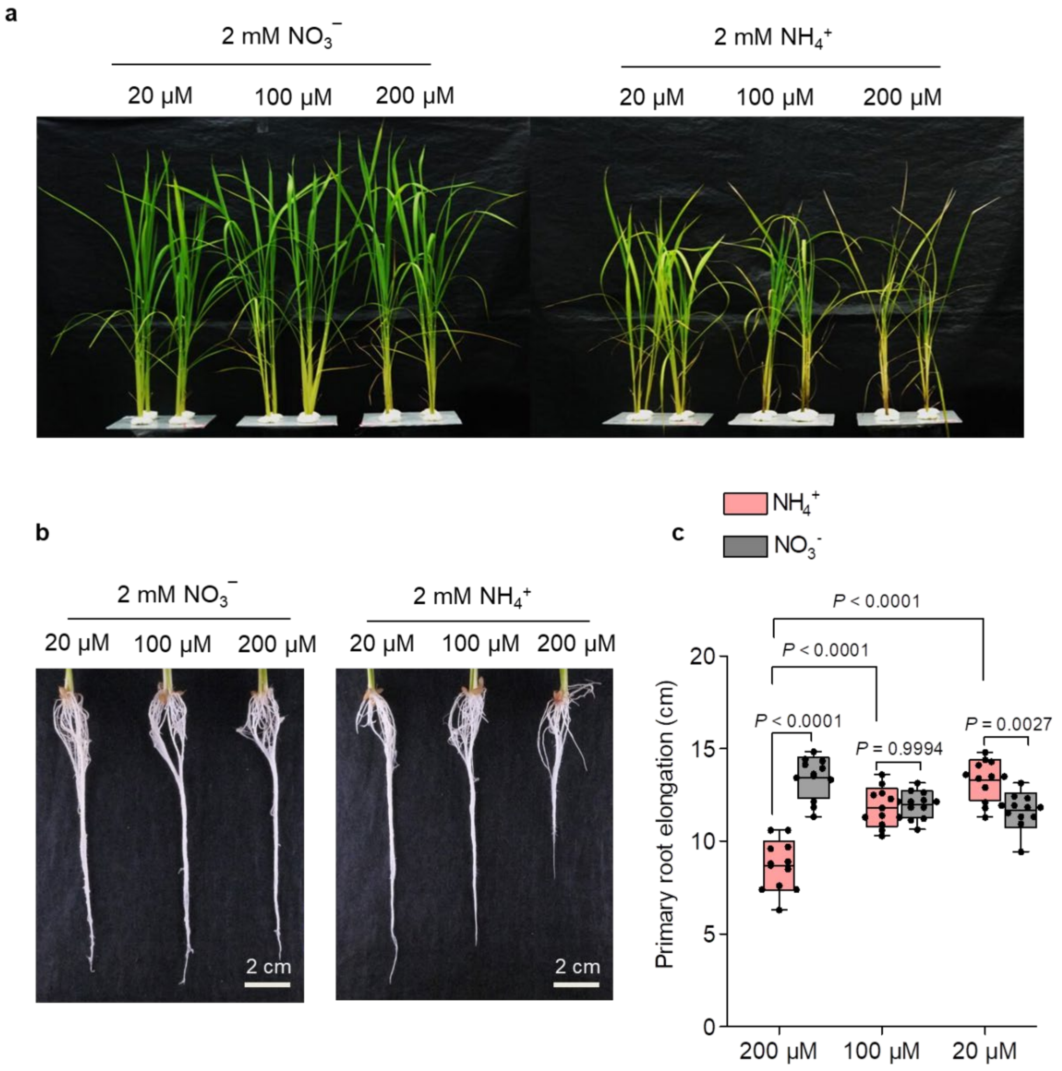


**Supplementary Figure 11 Growth response of rice plants (*Oryza sativa* [cv. Nipponbare]) in $NO_3^-$ or $NH_4^+$ medium with various doses of Fe.** Ten-day-old rice seedlings were transferred to $NO_3^-$ or $NH_4^+$ medium with various doses of Fe and analyzed 7 d after seedling transfer. **a** Shoot phenotype. **b, c** Root growth. Centre line represents mean and bounds of box are standard deviation; whiskers indicate the minimum and maximum values. *P*-values < 0.05 indicate significant differences (two-way ANOVA with post-hoc Tukey HSD test; n = 12 seedlings per condition). The experiment was repeated independently three times with similar results, and a representative experiment is shown.

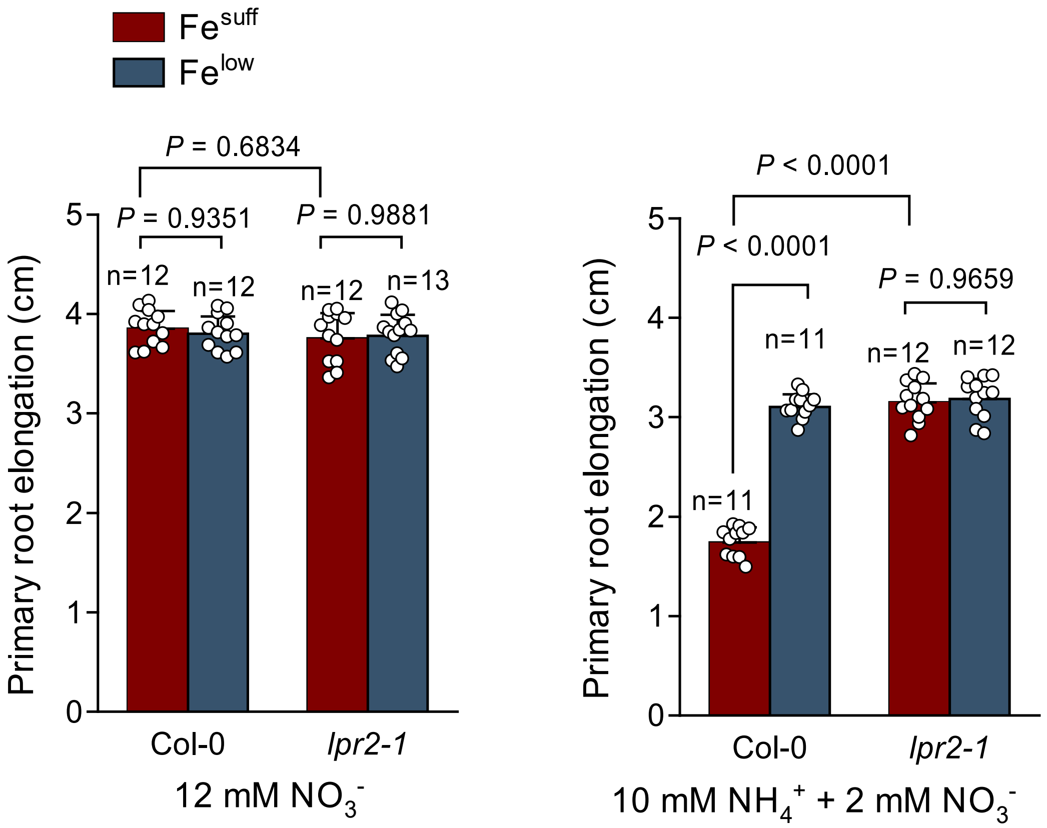


**Supplementary Figure 12 Growth response of primary roots of Col-0 and *lpr2-1* seedlings in high $NH_4^+/NO_3^-$ ratio conditions.** Four-day-old seedlings were transferred to the indicated media with $Fe^{suff}$ (100 µM) or $Fe^{low}$ (10 µM) and analyzed 4 d after seedling transfer. Data shown are means ± SD. n = number of seedlings. *P*-values < 0.05 indicate significant differences (two-way ANOVA with post-hoc Tukey HSD test). The experiment was repeated independently three times with similar results, and a representative experiment is shown.

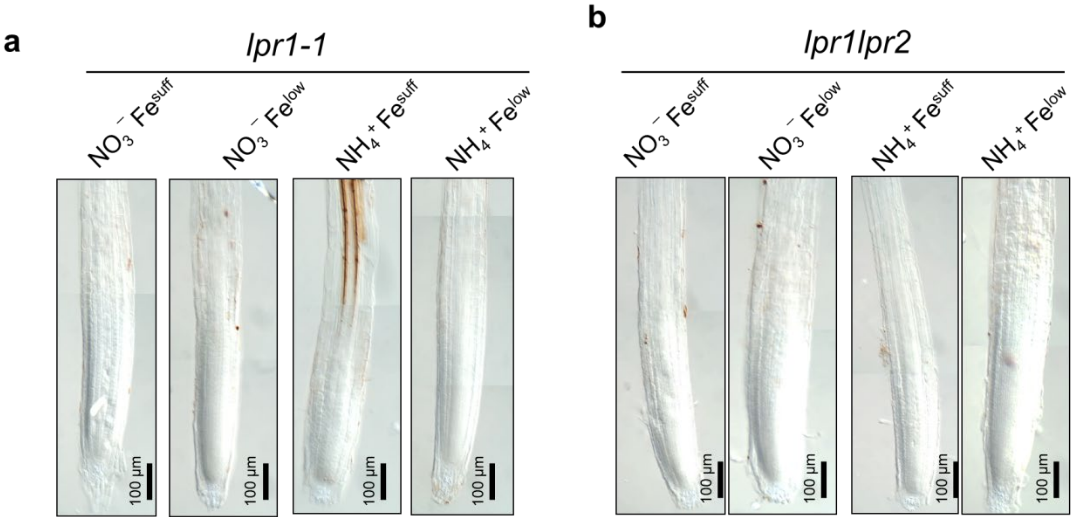


**Supplementary Figure 13 Perls/DAB staining in primary roots of *lpr1-1* and *lpr1lpr2* seedlings*.*** Four-day-old seedlings of the indicated genotype were transferred to $NO_3^-$ or $NH_4^+$ medium with $Fe^{suff}$ (100 μM) or $Fe^{low}$ (10 μM), and Perls/DAB staining was performed 4 d after seedling transfer. The experiment was repeated independently three times with similar results, and a representative experiment is shown.

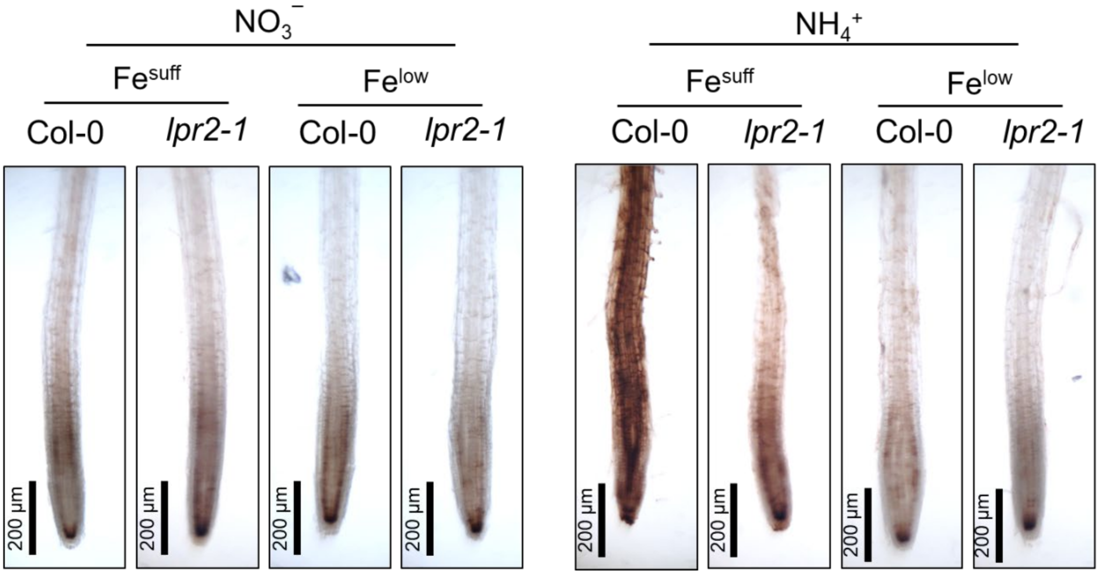


**Supplementary Figure 14 Fe deposition in primary roots of Col-0 and *lpr2-1* seedlings grown in light-irradiated medium.** Four-day-old seedlings were transferred to light-irradiated rooting medium with $NO_3^-$ or $NH_4^+$ as the nitrogen source and supplemented with either $Fe^{suff}$ (100 µM) or $Fe^{low}$ (10 µM). Analyses were performed 4 d after seedling transfer. The experiment was repeated independently three times with similar results, and a representative experiment is shown.

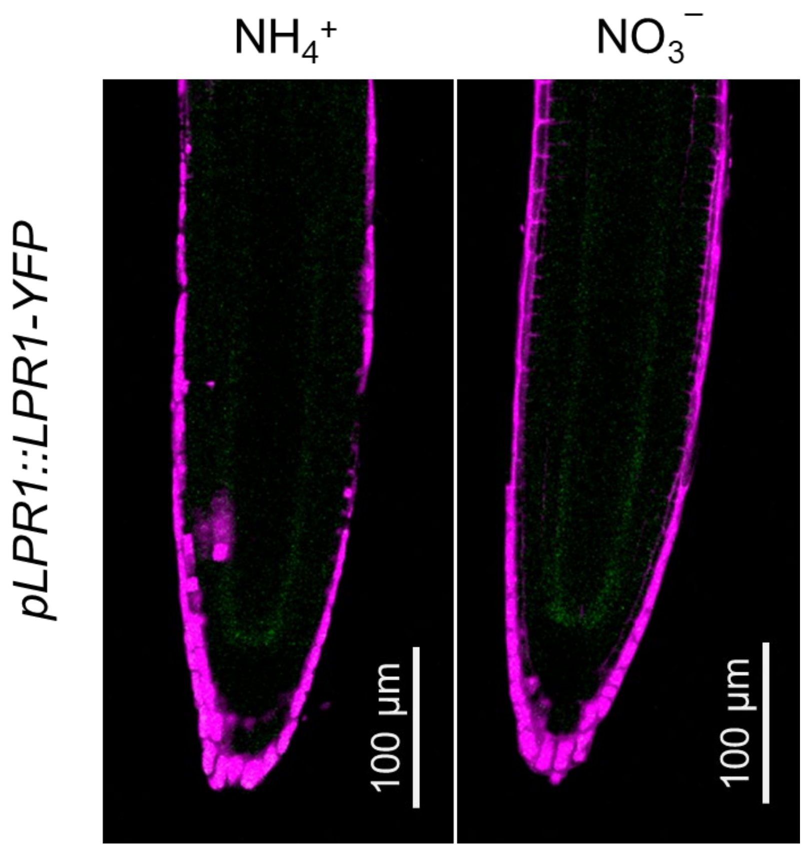


**Supplementary Figure 15 *pLPR1::LPR1-YFP* expression in primary roots.** Four-day-old *pLPR1::LPR1-YFP*/*lpr1-1* seedlings were transferred to $NO_3^-$ or $NH_4^+$ medium with $Fe^{suff}$ (100 µM) and analyzed 4 d after seedling transfer. The experiment was repeated independently three times with similar results, and a representative experiment is shown.

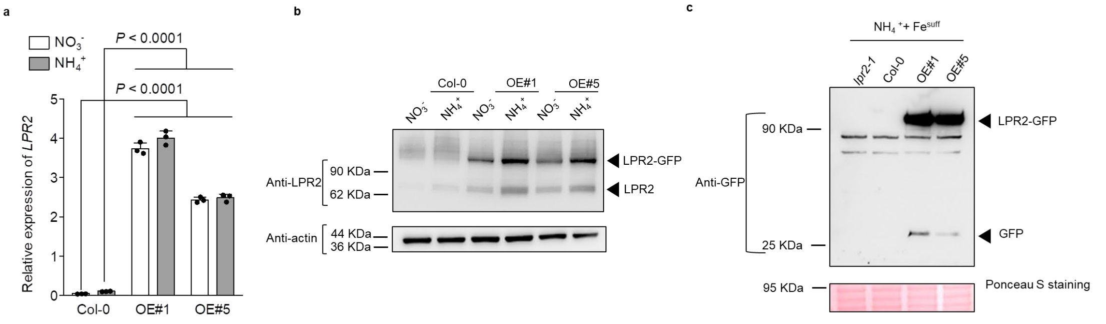


**Supplementary Figure 16 Characterization of *p35::LPR2-GFP* overexpression lines. a** *LPR2* expression in roots. Data shown are mean ± SD of three biological replicates. *P*-values < 0.05 indicate significant differences (two-way ANOVA with post-hoc Tukey HSD test). **b** Immunoblot for LPR2 in roots. **c** Immunoblot of GFP in roots. The signals of free GFP in two *p35::LPR2-GFP* overexpression lines suggest a cleavage of the LPR2-GFP protein. Four-day-old seedlings of the indicated genotypes were transferred to medium with $NO_3^-$ or $NH_4^+$ as the nitrogen source and 100 μM Fe supplementation and analyzed 4 d after seedling transfer. The experiment was repeated independently twice with similar results, and a representative experiment is shown.

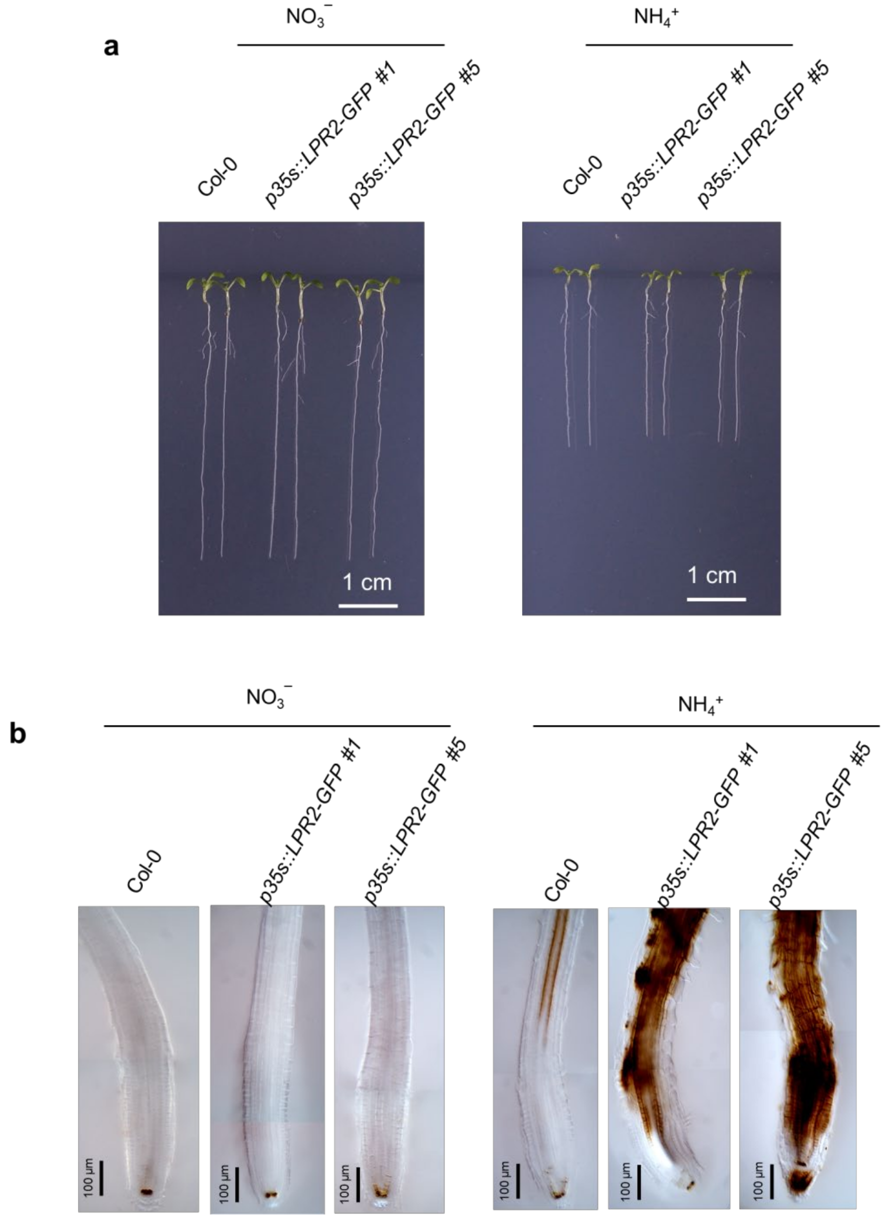


**Supplementary Figure 17 Effects of LPR2 overexpression on $NH_4^+$ sensitivity and Fe deposition in primary roots. a** Comparison of $NH_4^+$ sensitivity in Col-0 and *p35s::LPR2-GFP* overexpression lines. **b** Fe deposition indicated by Perls/DAB staining in primary roots. Four-day-old seedlings of the indicated genotypes were transferred to $NO_3^-$ or $NH_4^+$ medium with $Fe^{suff}$ (100 μM) and analyzed 4 d after seedling transfer. Each experiment was repeated independently twice with similar results, and a representative experiment is shown.

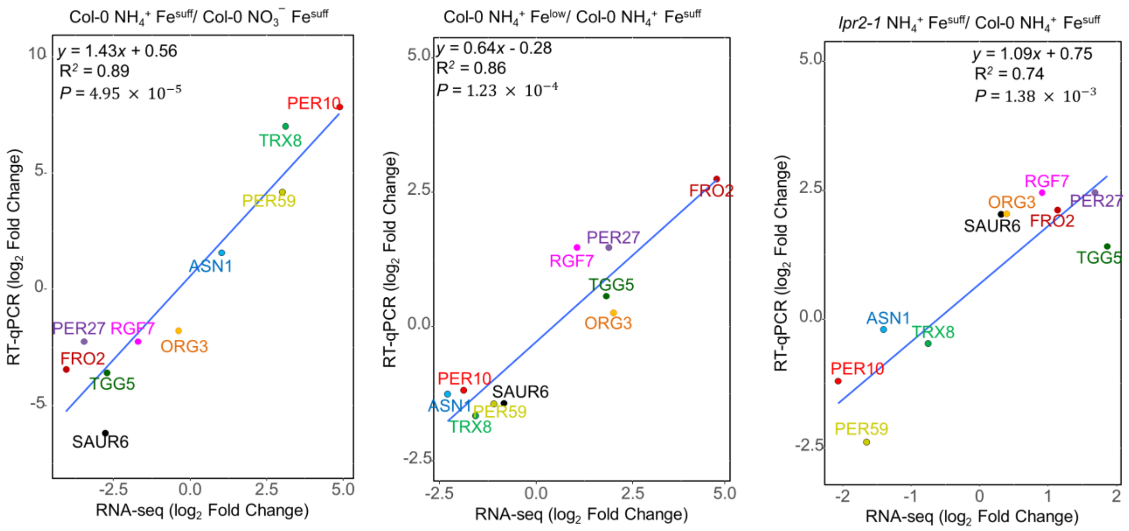


**Supplementary Fig. 18 Validation by RT-qPCR of the expression patterns of differentially expressed genes selected from the RNA-Seq analysis.** Four-day-old seedlings of the indicated genotype were transferred to $NO_3^-$ or $NH_4^+$ medium with $Fe^{suff}$ (100 μM) or $Fe^{low}$ (10 μM). Three biological replicates per line and condition were used for both RNA sequencing and RT-qPCR assays. The *P*-value reported for the linear regression is the regression tool ANOVA table's F-test results. RNA sequencing and RT-qPCR were conducted 4 d after seedling transfer.

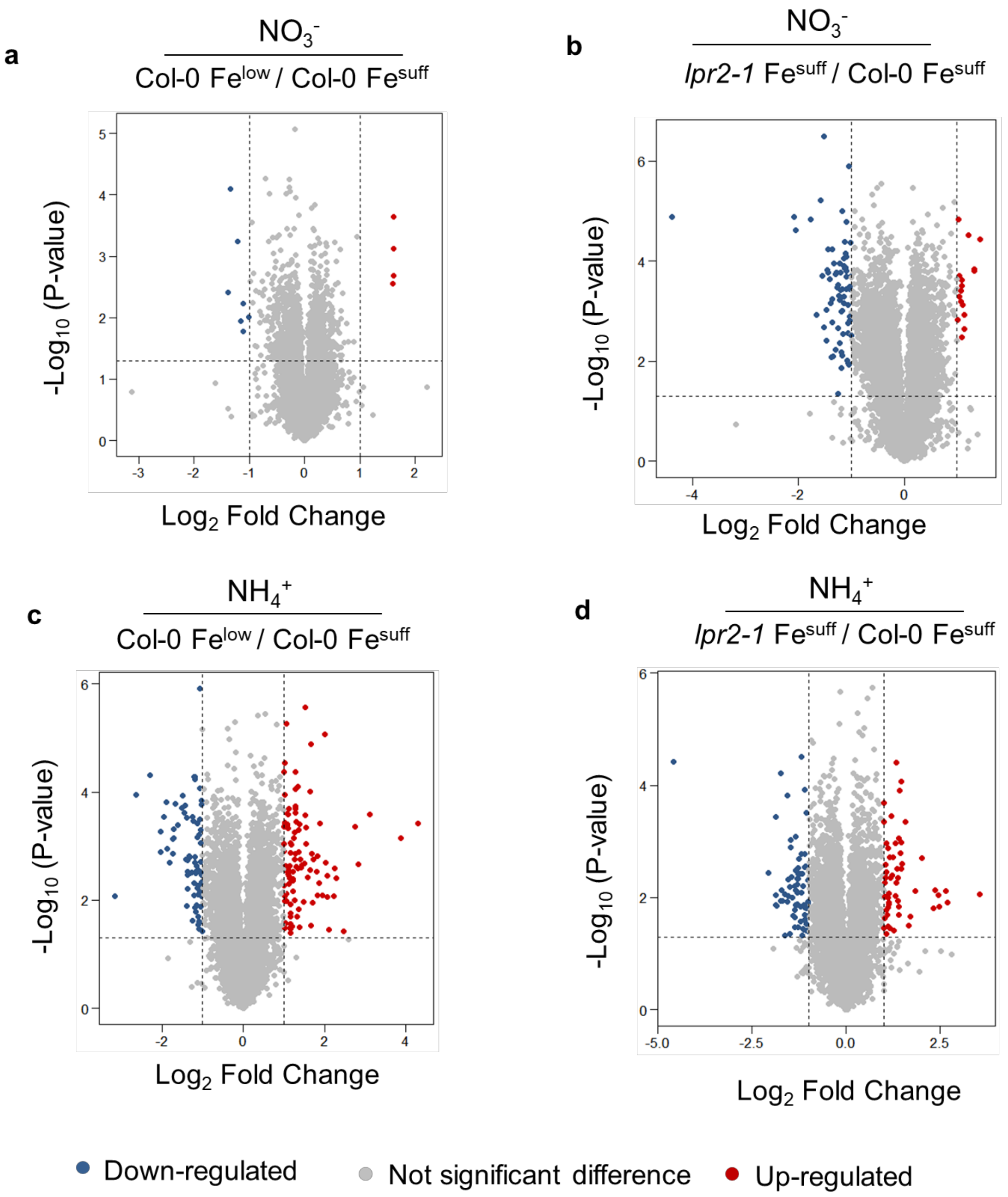


**Supplementary Figure 19 Volcano plots of the differentially expressed genes (DEGs). a** Volcano plot of DEGs between two Fe treatments for Col-0 seedlings in the $NO_3^-$-N source. **b** Volcano plot of DEGs between two genotypes given $Fe^{suff}$ treatment in the $NO_3^-$-N source. **c** Volcano plot of DEGs between two Fe treatments for Col-0 seedlings in the $NH_4^+$-N source. **d** Volcano plot of DEGs between two genotypes given $Fe^{suff}$ treatment in the $NH_4^+$-N source. The x-coordinate represents $|\log_2(\text{fold change})|$, and the y-coordinate represents $-\log_{10}(P\text{-value})$. The *P*-value for differential

expression was calculated using a parametric F-test comparing nested linear models. Each dot represents a gene. Red dots represent upregulated genes with significant expression. Blue dots represent downregulated genes with significant expression. Black dots represent genes with nonsignificant difference in expression. Four-day-old seedlings of the indicated genotypes were transferred to $NO_3^-$ or $NH_4^+$ medium with $Fe^{suff}$ (100 μM) or $Fe^{low}$ (10 μM) and analyzed 4 d after seedling transfer. The RNA sequencing experiment was conducted with three biological replicates per line and condition.

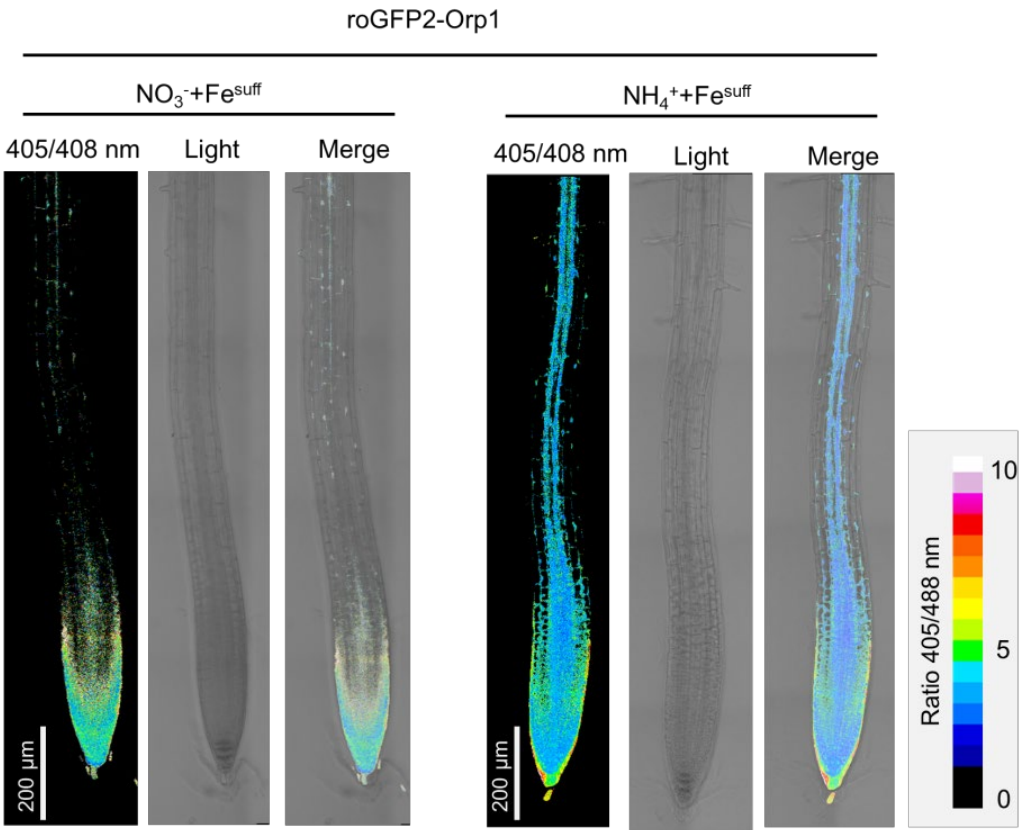


**Supplementary Figure 20 False color representation of $H_2O_2$ in primary roots of roGFP2-Orp1 seedlings.** Four-day-old roGFP2-Orp1 seedlings were transferred to $NO_3^-$ or $NH_4^+$ medium with $Fe^{suff}$ (100 µM). The oxidation state of roGFP2-Orp1 (ratio 400/485 nm) was analyzed 4 d after seedling transfer. The experiment was repeated independently three times with similar results, and a representative experiment is shown.

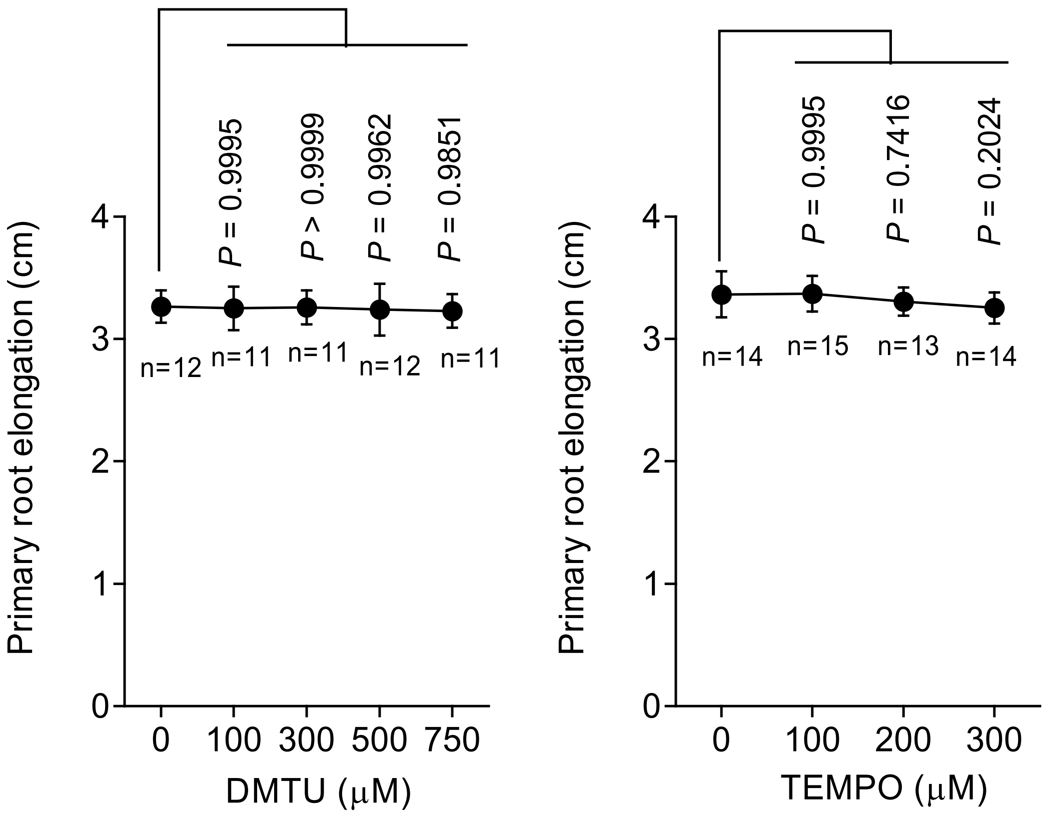


**Supplementary Figure 21 Effects of reactive oxygen species scavengers on the primary root growth of Col-0 seedlings grown in $NO_3^-$ medium.** Four-day-old Col-0 seedlings were transferred to $NO_3^-$ medium with $Fe^{suff}$ (100 μM) and various doses of indicated scavengers. Data shown are means ± SD. n = number of seedlings. *P*-values > 0.05 indicate nonsignificant differences (one-way ANOVA with post-hoc Tukey HSD test). The analyses were performed 4 d after seedling transfer. The experiment was repeated independently three times with similar results, and a representative experiment is shown.

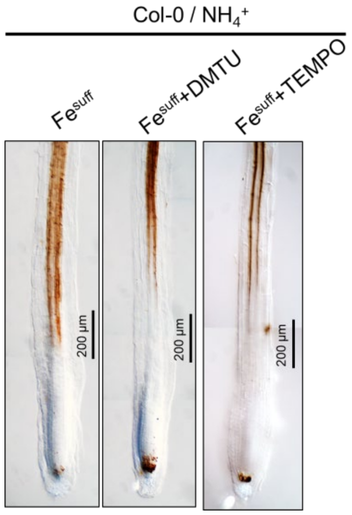


**Supplementary Figure 22 Effect of reactive oxygen species scavengers on Fe deposition in primary roots of Col-0 seedlings supplemented with $NH_4^+$.** Four-day-old Col-0 seedlings were transferred to $NH_4^+$ medium with $Fe^{suff}$ (100 µM), and Perls/DAB staining was performed 4 d after seedling transfer. The experiment was repeated independently three times with similar results, and a representative experiment is shown.

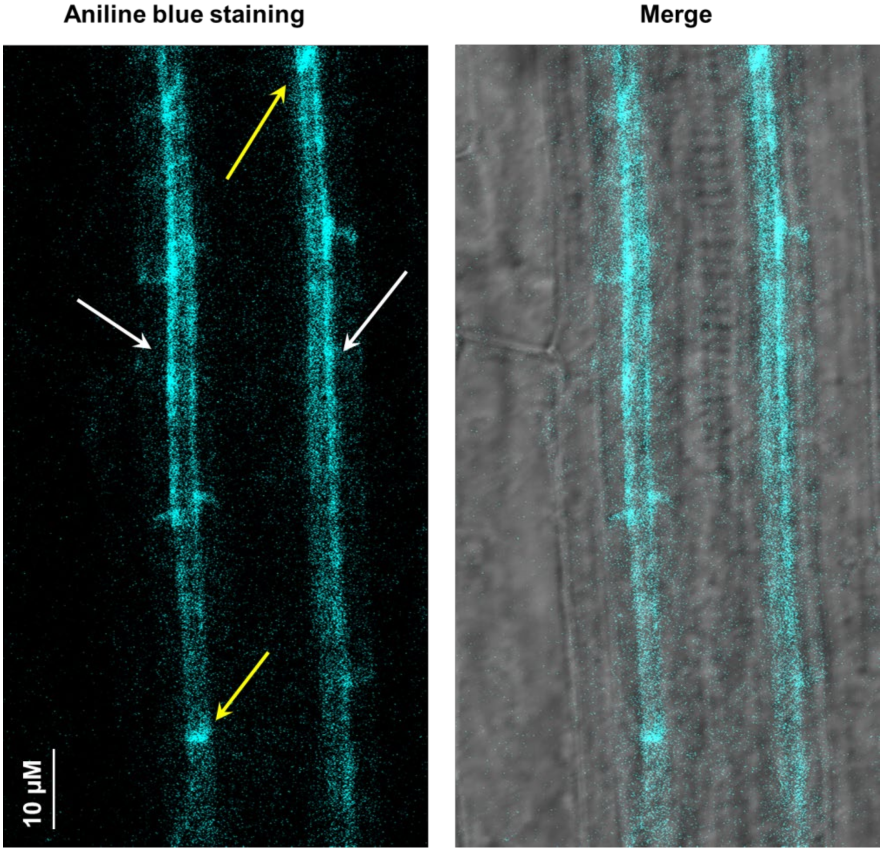


**Supplementary Figure 23 Callose detection in the phloem of the primary roots of Col-0 seedlings grown in $Fe^{suff}$ $NH_4^+$ medium.** Four-day-old Col-0 (WT) seedlings were transferred to $NH_4^+$ medium with $Fe^{suff}$ (100 μM), and aniline blue staining was performed 4 d after seedling transfer. Glycerol was used as the immersion medium for root samples to improve the resolution of confocal imaging. White and yellow arrows indicate callose deposition at lateral cell walls and sieve plates of the phloem, respectively. The experiment was repeated independently three times with similar results, and a representative experiment is shown.

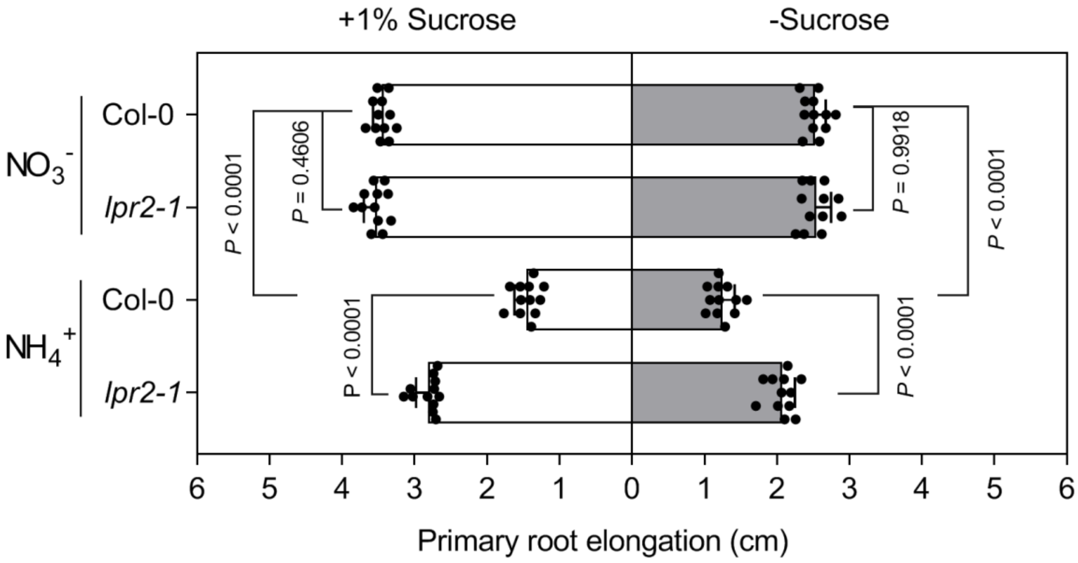


**Supplementary Figure 24 Growth response of primary roots of intact seedlings in media with or without 1% sucrose.** Four-day-old seedlings of the indicated genotypes were transferred to $NO_3^-$ or $NH_4^+$ medium with or without $Fe^{suff}$ (100 µM) and analyzed 4 d after seedling transfer. Data shown are means ± SD of 12 seedlings per line and condition. *P*-values < 0.05 indicate significant differences (two-way ANOVA with post-hoc Tukey HSD test). The experiment was repeated independently three times with similar results, and a representative experiment is shown.

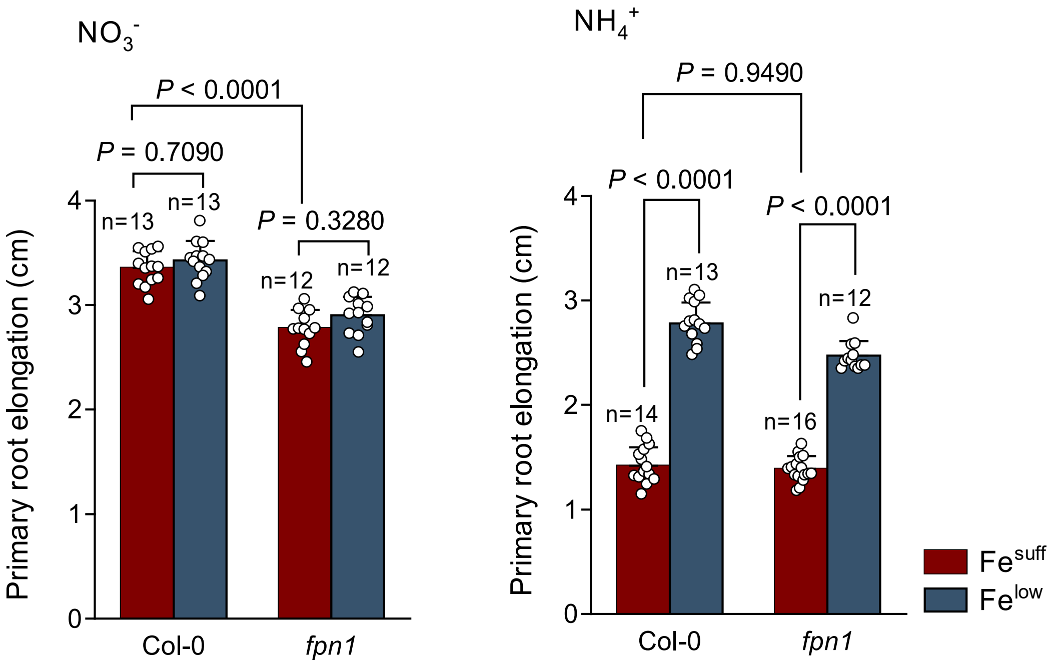


**Supplementary Figure 25 Growth response of primary roots of *fpn1* mutants in $NH_4^+$ medium.** Four-day-old Col-0 and *fpn1* seedlings were transferred to $NO_3^-$ or $NH_4^+$ medium with $Fe^{suff}$ (100 µM) and analyzed 4 d after seedling transfer. Data shown are means ± SD of three biological replicates. *P*-values < 0.05 indicate significant differences (two-way ANOVA with post-hoc Tukey HSD test; n = number of seedlings). The experiment was repeated independently three times with similar results, and a representative experiment is shown.
