# Supplementary material for: Phloem iron remodels root development in response to ammonium as the major nitrogen source
Source: Nat Commun. 2022 Jan 28;13:561. doi: 10.1038/s41467-022-28261-4 (PMC8799741; doi:10.1038/s41467-022-28261-4)
Supplement: Supplementary file 3 — Description of Additional Supplementary Files [file 41467_2022_28261_MOESM3_ESM.pdf]

Description of Additional Supplementary Files

File: Supplementary Data 1

Description: RNA-seq data sets and lists of differentially expressed genes (DEGs). **a** Summary of RNA-seq data sets. **b** List of DEGs between Fe^{low} and Fe^{suff} treatments in Col-0 under NO_3^- supply. **c** List of DEGs between *lpr2-1* and Col-0 in Fe^{suff} treatment under NO_3^- supply. **d** List of DEGs between Fe^{low} and Fe^{suff} treatments in Col-0 under NH_4^+ supply. **e** List of DEGs between *lpr2-1* and Col-0 in Fe^{suff} treatment under NH_4^+ supply. **f** Expression of the genes related to NH_4^+ toxicity.

File name: Supplementary Data 2

Description: Oligonucleotide Primers Used in This Study.
