# Supplementary material for: Phloem iron remodels root development in response to ammonium as the major nitrogen source
Source: Nat Commun. 2022 Jan 28;13:561. doi: 10.1038/s41467-022-28261-4 (PMC8799741; doi:10.1038/s41467-022-28261-4)
Supplement: Supplementary file 6 — Reporting Summary [file 41467_2022_28261_MOESM6_ESM.pdf]

nature portfolio

Corresponding author(s): Chong Wei Jin

Last updated by author(s): Dec 18, 2021

Reporting Summary

Nature Portfolio wishes to improve the reproducibility of the work that we publish. This form provides structure for consistency and transparency in reporting. For further information on Nature Portfolio policies, see our Editorial Policies and the Editorial Policy Checklist.

Statistics

For all statistical analyses, confirm that the following items are present in the figure legend, table legend, main text, or Methods section.

n/a	Confirmed	
☐	☒	The exact sample size (n) for each experimental group/condition, given as a discrete number and unit of measurement
☐	☒	A statement on whether measurements were taken from distinct samples or whether the same sample was measured repeatedly
☐	☒	The statistical test(s) used AND whether they are one- or two-sided<br>*Only common tests should be described solely by name; describe more complex techniques in the Methods section.*
☒	☐	A description of all covariates tested
☒	☐	A description of any assumptions or corrections, such as tests of normality and adjustment for multiple comparisons
☐	☒	A full description of the statistical parameters including central tendency (e.g. means) or other basic estimates (e.g. regression coefficient) AND variation (e.g. standard deviation) or associated estimates of uncertainty (e.g. confidence intervals)
☐	☒	For null hypothesis testing, the test statistic (e.g. F, t, r) with confidence intervals, effect sizes, degrees of freedom and P value noted<br>*Give P values as exact values whenever suitable.*
☒	☐	For Bayesian analysis, information on the choice of priors and Markov chain Monte Carlo settings
☒	☐	For hierarchical and complex designs, identification of the appropriate level for tests and full reporting of outcomes
☒	☐	Estimates of effect sizes (e.g. Cohen's d, Pearson's r), indicating how they were calculated

Our web collection on statistics for biologists contains articles on many of the points above.

Software and code

Policy information about availability of computer code

Data collection	Signal peptide was predicted by SignalP 5.0 algorithm. The Km and Vmax were calculated using the Michaelis–Monod model in the GraphPad Prism 8 software. Confocal images were collected using Olympus FV3000 and Zeiss LSM 880. qRT-PCR data were collected using StepOnePlus Software Ver. 2.2.3 (Agilent). Sequencing reader were collected using Hiseq Xten sequencer (Illumina).
Data analysis	1. The RNA-seq data were analyzed analyzed using Stringtie and ballgown. Significant DEGs were assessed using DEGseq. GO term enrichment analysis was performed with R packages clusterProfiler and database org.At.tair.db (version 3.13.0).<br>2. All statistics for the other quantitative results were analyzed using the Prism v8 (GraphPad, San Diego, CA, USA).

For manuscripts utilizing custom algorithms or software that are central to the research but not yet described in published literature, software must be made available to editors and reviewers. We strongly encourage code deposition in a community repository (e.g. GitHub). See the Nature Portfolio guidelines for submitting code & software for further information.

Data

Policy information about availability of data

All manuscripts must include a data availability statement. This statement should provide the following information, where applicable:

- Accession codes, unique identifiers, or web links for publicly available datasets
- A description of any restrictions on data availability
- For clinical datasets or third party data, please ensure that the statement adheres to our policy

The authors declare that the data supporting the findings of this study are available within the paper, the Supplementary Information, and the Source Data file.

Field-specific reporting

Please select the one below that is the best fit for your research. If you are not sure, read the appropriate sections before making your selection.

☒ Life sciences ☐ Behavioural & social sciences ☐ Ecological, evolutionary & environmental sciences

For a reference copy of the document with all sections, see nature.com/documents/nr-reporting-summary-flat.pdf

Life sciences study design

All studies must disclose on these points even when the disclosure is negative.

Sample size	Required experimental sample size were determined based on previous studies in the field to enable statistical analysis and ensure reproducibility.
Data exclusions	No data were excluded from the analysis.
Replication	Sample sizes have been increased wherever possible. All attempts at replication were successful.
Randomization	Samples were randomly collected.
Blinding	This was not practical given our design.

Reporting for specific materials, systems and methods

We require information from authors about some types of materials, experimental systems and methods used in many studies. Here, indicate whether each material, system or method listed is relevant to your study. If you are not sure if a list item applies to your research, read the appropriate section before selecting a response.

Materials & experimental systems

n/a	Involved in the study	
☐	☒	Antibodies
☒	☐	Eukaryotic cell lines
☒	☐	Palaeontology and archaeology
☒	☐	Animals and other organisms
☒	☐	Human research participants
☒	☐	Clinical data
☒	☐	Dual use research of concern

Methods

n/a	Involved in the study	
☒	☐	ChIP-seq
☒	☐	Flow cytometry
☒	☐	MRI-based neuroimaging

Antibodies

Antibodies used	Custom-made anti-LPR2 antibody produced in rabbits (ABclonal), Mouse monoclonal anti-Actin (Sangon Biotech, D191048), rabbit polyclonal anti-GFP (ABclonal, AE011). HRP-conjugated goat anti-rabbit IgG (Sangon Biotech, D110058), HRP-conjugated goat anti-mouse IgG (Sangon Biotech, D110087).
Validation	The custom-made anti-LPR2 antibody was validated by detecting LPR2 protein in wild-type (Col-0), lpr2 mutants and p35s::LPR2 overexpression lines. Validation of all the other commercial antibodies could be found on the manufacturers' websites.
