# Supplementary material for: Phloem iron remodels root development in response to ammonium as the major nitrogen source
Source: Nat Commun. 2022 Jan 28;13:561. doi: 10.1038/s41467-022-28261-4 (PMC8799741; doi:10.1038/s41467-022-28261-4)
Supplement: Supplementary file 2 — Peer Review File [file 41467_2022_28261_MOESM2_ESM.pdf]

nature portfolio

Peer Review File

Phloem iron remodels root development in response to ammonium as the major nitrogen source

Open Access This file is licensed under a Creative Commons Attribution 4.0 International License, which permits use, sharing, adaptation, distribution and reproduction in any medium or format, as long as you give appropriate credit to the original author(s) and the source, provide a link to the Creative Commons license, and indicate if changes were made. In the cases where the authors are anonymous, such as is the case for the reports of anonymous peer reviewers, author attribution should be to 'Anonymous Referee' followed by a clear attribution to the source work. The images or other third party material in this file are included in the article's Creative Commons license, unless indicated otherwise in a credit line to the material. If material is not included in the article's Creative Commons license and your intended use is not permitted by statutory regulation or exceeds the permitted use, you will need to obtain permission directly from the copyright holder. To view a copy of this license, visit http://creativecommons.org/licenses/by/4.0/.

<b>REVIEWER COMMENTS</B>

Reviewer #1 (Remarks to the Author):

Nitrogen is one of the essential plant nutrients required in substantial amounts to maintain adequate plant growth. It is mainly acquired by plant roots in the form of ammonium and nitrate. Nevertheless, these two forms of nitrogen sources have distinct impacts on root development. The manuscript by Liu et al. proposed a novel biologically interesting mechanism, namely that iron deposition in the phloem apoplast regulates the morphological responses of plant root to different nitrogen sources. The authors first screened a T-DNA insertion mutant which is insensitive to ammonium stress. The T-DNA insertion in this mutant was annotated to be in the ferroxidase gene LPR2. Subsequently, they identified that, in comparison with nitrate as the nitrogen source, the ammonium upregulates LPR2 in the cell wall of the root stele. This action results in excessive iron deposition in phloem apoplast via a catalytic reaction of Fe^{2+} oxidation to Fe^{3+}, which triggers ROS burst and consequently leads to massive callose deposition. As a result, sucrose is insufficiently allocated to the root growth zone due to inhibition of phloem action, and thus causes stunted growth of the primary root.

The collection of data sets is impressive and details the process of discovery in relating the action of phloem iron to root development. For the most part the manuscript is well written and concise. The comments and suggested changes below are aimed at helping them present a more complete story.

Major points

1. The line number of the MS should be added, otherwise it is difficult to locate.

2. Abstract

The methods used in the present study should also be added in Abstract

3. It is reported that H^+-ATPase was also involved in NH_4^+ tolerance in rice (Zhu et al., 2009; Weng et al., 2020). I think those results should be added in Introduction section.

4. The root growth of lpr2 mutants showed higher tolerance to ammonium stress. The plant tolerance to ammonium stress may be associated with the uptake of ammonium by roots and its accumulation in plant tissue. Therefore, evidences should be provided to clarify whether the tolerant phenotype of lpr2 mutants is associated with a decreased root ammonium uptake or a reduced ammonium level in plants.

5. The authors showed that ammonium condition led to greater up-regulation of LPR2 activity in the root stele than nitrate, and proposed that this up-regulation is a part mechanism for the LPR2-mediated ammonium-sensitive phenotype. However, I have not seen the phenotype of the LPR2 overexpression lines in ammonium conditions. Am I missing it? It might be a piece of positive evidence if LPR2-OE lines showed more sensitive to ammonium stress. Besides, expression of the LPR2-YFP of complementary lines in Fig. 1D should be tested in a qRT-PCR for the same reason.

6. The pH buffer MES is present in all the treatments. This is ok and I also encourage to addition of pH buffer in the growth medium, because the natural soil growth conditions have a pH buffer capacity. Nevertheless, I'm still curious whether the lpr2 mutants also have an ammonium-tolerant phenotype in the medium without pH buffer MES.

7. How many times was each experiment carried out? This should be explicitly indicated in the M&M or figure legends.

Minor points:

1. Lines: “Considering the phloem cell-specific Fe deposition in response to NH4+ ……” and “A close-up view of H2DCFDA-stained roots revealed that the NH4+-triggered……”, NH4+ should be revised to NH4+, and H2DCFDA should be revised to H2DCFDA. Check throughout.

2. Line: “Upon 3-d gradual elevation of sucrose from 1–3% or more in the upper layer under Fesuff NH4+ conditions……”, the statement does not accurately describe the experiment shown in Fig. 7C

3. Line in methods “clustering and selected for subsequent GO analysis by using R package clusterProfiler.” is an incorrect statement, because R package cannot perform a GO Enrichment Analysis.

4. Line in methods “GFP/YFP/H2CDFA ex: 488 nm, em:500–550 nm; PI/FM4-64 ex: 561, em: 570–670” need to add a space.

5. Line in methods “A P <0.05 was considered significant” should be revised.

6. The scale bar is incorrectly marked in Fig. 1B.

7. Fig. 3E and 3F: The meaning of the red arrow and green arrow needs to be indicated in the figure legend.

8. Statistics should be done in Fig. 5F and Fig.5G.

9. One scale bar is missed in Fig.7B.

10. Since no Fe deposition was observed in Suppl. Fig. 7, the title “Fe deposition in primary roots of lpr1lpr2” of this Figure is not exact.

Reviewer #2 (Remarks to the Author):

The manuscript by Liu et al. analyzes a signal mechanism leading to ammonium-induced primary root growth inhibition. They identified a mutant which alleviated ammonium-induced primary root growth inhibition, and which they identified as the ferroxidase LPR2. Its homologue, LPR1 was shown to be crucial player in phosphate deficiency-induced primary root growth inhibition, but a precise role for LPR2 has not been shown yet. In analogy to LPR1, the authors convincingly show, that the cell wall localized LPR2 is involved in ammonium-induced ROS accumulation and callose deposition. They further show that the accumulation of iron, which is the substrate of LPR2, is crucial for ammonium-induced primary root growth inhibition. As such, they provide a model, in which high levels of ammonium triggers apoplastic accumulation of iron, which is oxidized by the action of LPR2, leading to the accumulation of ROS and subsequently to the accumulation of callose. Unlike for the situation in phosphate deficiency, in which these events have been described to occur in the stem cell niche, for ammonium toxicity, these events take place in the differentiation zone of the root, specifically in the phloem. As such, the model is extended by the observation that callose deposition in the phloem impairs its function so that transport of factors with high importance for cellular integrity (such as sucrose) cannot be transported to the lower part of the roots, leading to early differentiation and primary root growth inhibition.

The results are very interesting and novel and will attract the attention of many scientists working on stress signal transduction. In general, the experiments are properly conducted yielding convincing results, which also justify for most parts the claims by the authors. However, there are some points which should be addressed in order to improve an already good manuscript, which are detailed below.

General major comments:

The rescue of ammonium-induced primary root growth inhibition was shown for the mutants lpr2-1 and isas. It is also shown that the WT phenotype can be restored by complementation of the lpr2-1 mutant by expressing LPR2-YFP under control of the LPR2 promoter. However, all subsequent experiments (iron

and callose accumulation, ROS staining) were performed only with lpr2-1. It would strengthen the manuscript, if these experiments would be conducted additionally with at least the complementation line as well.

Additionally, since the lpr2-1 mutant can be complemented by an pLPR2::LPR2-YFP construct, YFP tagged LPR2 seem to be active. As such, it is reasonable, that in the p35S::LPR2-GFP plants, which they used for subcellular localization of LPR2, LPR2 is also active. Therefore, the authors also have generated a stable LPR2 overexpression line. However, nothing beside the subcellular localization of LPR2 is mentioned. It would be interesting to see, if these LPR2 overexpression lines are hypersensitive with respect to ammonium-induced primary root growth inhibition, iron and callose deposition and ROS accumulation. If not, this would mean that additional factors (such as the discussed availability of the LPR2 substrate Fe) are important factors in the signaling of ammonium-induced primary root growth inhibition.

Also, the text should undergo an English spelling and grammar check.

Specific comments:

Introduction: The introduction is very short and not very informative. The authors should mention more about the current state of ammonium toxicity research, i.e. detoxification mechanisms, uptake etc. Also, current knowledge about the signaling events taking place in ammonium-induced primary root growth inhibition should be mentioned, if such information is available.

Material and Methods:

1. AGI codes should be added to Suppl Table 2 for each gene

2. Considering the source of the mutants, the authors can already refer to the gene model shown in Figure 1A, in which the location of primers used for genotyping and qRT-PCRs should be indicated as well.

3. Could the authors present a schematic drawing how they measured root meristem size?

4. Which method was used for calculation of expression values measured by qRT-PCR?

5. How was quantification of band intensities for Fig 4B performed?

Results/Discussion:

1. Are the mutants lpr2-1 and isas knock-outs or knock downs? LPR2 Expression levels of all mutants and the complementation and overexpression line should be shown. Also, western blots similar to the one shown in Figure 4B should be shown for both mutants, the complementation and the overexpression lines.

2. Fig 2: Although cell wall localization is plausible according to the picture shown in Fig 2., the quality of the picture should be improved. There are several examples in the literature, in which plasmolysis was much more evident compared to Figure 2B, which would strengthen the argument that LPR2 is indeed

localized to the cell wall. Additionally, are there any hints about the presence of a signal peptide in LPR2 (such as in LPR1)?

3. Fig 2C and D: There is some confusion in the legend. “Data shown are means +-SD of three biological replicates” refer to Fig 2D, not to Fig 2C.

4. Fig 2 C and D: It would have been biochemically more relevant, if the GST tag would have been removed. However, this is not a major concern, since the results clearly show that LPR2 is a ferroxidase, which is the major take-home message. However, the authors discuss that (Page 15) “… their enzymatic behaviours are different”. This statement is not valid, since the authors could not show any activity for LPR1. Since native LPR1 has been characterized as a ferroxidase (Reference #20), its activity is probably impaired by the GST tag. Therefore, the authors should refer more to Ref #20 for LPR1 enzyme characteristics and compared it to their LPR2 enzyme characteristics. Also, how were Km and vmax values calculated?

5. Fig 2E: what was the LPR1 expression level in the pLPR2::LPR1 plants? Was it comparable to LPR2 expression levels in WT?

6. Fig 3: In contrast to the statement in the last paragraph of page 6, Fe deposition is not shown for lpr1-1.

7. Is there an explanation for the higher Fe content in the stem cell niche in WT roots exposed to low Fe compared to high Fe?

8. Fig 3 Legend: the abbreviations and the meaning of the arrows should be defined.

9. Fig 5, RNA seq results: It is commonly accepted that some expression levels obtained by RNA seq should be confirmed by qRT-PCR. The authors should also provide a table showing the RNA seq data for all genes and not only for the DEGs. Although the intention of the comparisons between high and low Fe is clear, the authors should also provide results from other comparisons, such as between lpr2-1 and WT, which could be of strong interest to many readers. Also, the data should be discussed in more detail. Are there any differences in expression of genes related to ammonium toxicity (ammonium generation, detoxification, transport etc.) between the treatment / genotypes, especially in relation to the observed root growth phenotypes? The meaning of the scale bar (probably log2 fold) should be added to Fig 5a. What have been the conditions for the results shown in Figure 5E (probably Fe suff NH_4^+)? Page 11: Fig 5 G should read Fig 5 H.

10. Fig 6: Although not cell-specific, why does callose overaccumulate in lpr2-1 mutants? Considering the Fe-ROS-Callose correlation: The authors should discuss why callose accumulation in the stem cell niche of WT exposed to NH4 and Fe low is stronger compared to NO3 Fe low, while Fe accumulation is lower in WT exposed to NH4 Fe low compared to NO3 Fe low. It is hard to see from the pictures in Fig 6, but is there any preferential accumulation of callose at the sieve plates of the phloem, as has been observed for iron (Fig 3)? This might be important, since it is discussed on page 17.

11. Fig 7 D and E: A side by side comparison of the WT response to between NO3 and NH4 exposure at different sucrose levels would be more informative. Also, there is only one sucrose concentration (1 %) at which there is a difference, whereas at 2 and 3 %, there is hardly any difference between lpr2-1 and WT or between NO3- and NH4. Did the authors also try even lower sucrose concentrations?

12. Supplemental Figure 11: The meaning of the scale bar should be defined.

Discussion:

The discussion could be more comprehensive. For example: what could be the signaling events downstream of callose deposition leading to ammonium-induced primary root growth inhibition? Could it be that similar to phosphate deficiency, certain factors mediating cell fate, cannot be transported? What about signal molecules etc.?

REVIEWER COMMENTS

Reviewer #1 (Remarks to the Author):

Nitrogen is one of the essential plant nutrients required in substantial amounts to maintain adequate plant growth. It is mainly acquired by plant roots in the form of ammonium and nitrate. Nevertheless, these two forms of nitrogen sources have distinct impacts on root development. The manuscript by Liu et al. proposed a novel biologically interesting mechanism, namely that iron deposition in the phloem apoplast regulates the morphological responses of plant root to different nitrogen sources. The authors first screened a T-DNA insertion mutant which is insensitive to ammonium stress. The T-DNA insertion in this mutant was annotated to be in the ferroxidase gene LPR2. Subsequently, they identified that, in comparison with nitrate as the nitrogen source, the ammonium upregulates LPR2 in the cell wall of the root stele. This action results in excessive iron deposition in phloem apoplast via a catalytic reaction of Fe^{2+} oxidation to Fe^{3+}, which triggers ROS burst and consequently leads to massive callose deposition. As a result, sucrose is insufficiently allocated to the root growth zone due to inhibition of phloem action, and thus causes stunted growth of the primary root.

The collection of data sets is impressive and details the process of discovery in relating the action of phloem iron to root development. For the most part the manuscript is well written and concise. The comments and suggested changes below are aimed at helping them present a more complete story.

Response: Thank you very much for your positive comments on our manuscript.

Major points

1. The line number of the MS should be added, otherwise it is difficult to locate.

Response: We sincerely apologize for the inconvenience. The line number has been added in the revised manuscript.

2. The methods used in the present study should also be added in Abstract

Response: The key methods, including the knocking out of *LPR2* and reduction of iron supplementation, have been added to the Abstract.

3. It is reported that H^+-ATPase was also involved in NH_4^+ tolerance in rice (Zhu et al., 2009; Weng et al., 2020). I think those results should be added in Introduction section.

Response: The role of H^+-ATPase in NH_4^+ tolerance has been discussed in the Introduction section (lines 53-55).

4. The root growth of *lpr2* mutants showed higher tolerance to ammonium stress. The plant tolerance to ammonium stress may be associated with the uptake of ammonium by roots and its accumulation in plant tissue. Therefore, evidences should be provided to clarify whether the tolerant phenotype of *lpr2* mutants is associated with a decreased root ammonium uptake or a reduced ammonium level in plants.

Response: The uptake of NH_4^+ could result in acidification of the rhizosphere, which is suggested to be a mechanism for NH_4^+ toxicity. In our study, we used pH-buffered growth media to minimize rhizosphere acidification resulting from root NH_4^+ uptake. Therefore, the LPR2-mediated NH_4^+ sensitivity is not associated with rhizospheric acidification due to NH_4^+ uptake. Nevertheless, NH_4^+ uptake also affects cellular NH_4^+ concentration. It is generally held that NH_4^+ per se is a toxic compound. Therefore, we measured the NH_4^+ concentration in roots. However, the loss of LPR2 function did not affect the root NH_4^+ level (Supplementary Fig. 8), showing that LPR2-mediated NH_4^+ sensitivity is also unassociated with NH_4^+ accumulation.

5. The authors showed that ammonium condition led to greater up-regulation of LPR2 activity in the root stele than nitrate, and proposed that this up-regulation is a part mechanism for the LPR2-mediated ammonium-sensitive phenotype. However, I have not seen the phenotype of the LPR2 overexpression lines in ammonium conditions. Am I missing it? It might be a piece of positive evidence if LPR2-OE lines showed more sensitive to ammonium stress. Besides, expression of the LPR2-YFP of complementary lines in Fig. 1D should be tested in a qRT-PCR for the same reason.

Response: Based on the qRT-PCR and immunoblot assays, we found that the complementation lines had higher *LPR2* expression and LPR2 abundance in roots than those of Col-0 in the NH_4^+-N source with Fe^{suff} (Supplementary Fig. 5). This indicates that LPR2 was slightly overexpressed in the complementation lines.

However, the primary roots of these complementation lines had similar growth inhibition and Fe deposition in the NH_4^+-N source compared to those in Col-0 seedlings (Fig. 1). The PRs of *p35S::LPR2-GFP* overexpression lines also had a similar NH_4^+ sensitivity to those of Col-0 (Supplementary Fig. 17a). These results suggested that although the NH_4^+ sensitivity of PRs essentially depends on LPR2, additional factor(s) are also required for NH_4^+-induced PR growth inhibition. It is worth noting that the *p35S::LPR2-GFP* overexpression lines showed irregular Fe deposition in PRs in the NH_4^+-N source (Supplementary Fig. 17b), indicating that the NH_4^+-induced phloem-specific Fe deposition depends on the stele-specific distribution of LPR2.

In the revised manuscript, we have deleted the assumption that "up-regulation of LPR2 is a part mechanism for the LPR2-mediated ammonium-sensitive phenotype". Thank you for your valuable suggestion.

6. The pH buffer MES is present in all the treatments. This is ok and I also encourage to addition of pH buffer in the growth medium, because the natural soil growth conditions have a pH buffer capacity. Nevertheless, I'm still curious whether the *lpr2* mutants also have an ammonium-tolerant phenotype in the medium without pH buffer MES.

Response: As per your suggestion, the NH_4^+ sensitivity of *lpr2* mutants was also examined in un-buffered conditions and they still had better PR growth than Col-0 did in the NH_4^+-N source without MES (Supplementary Fig. 4). This result provides further support for the idea that the inhibitory effect of NH_4^+ on PR growth could be uncoupled by the loss of LPR2 function.

7. How many times was each experiment carried out? This should be explicitly indicated in the M&M or figure legends.

Response: The information on the independent replication of each experiment has been added to the figure legends.

Minor points:

1. Lines: "Considering the phloem cell-specific Fe deposition in response to

NH_4^+ ……” and “A close-up view of H2DCFDA-stained roots revealed that the NH_4^+-triggered……”, NH_4^+ should be revised to NH_4^+, and H2DCFDA should be revised to H2DCFDA. Check throughout.

Response: These have been revised and the spelling has been carefully checked throughout.

2. Line: “Upon 3-d gradual elevation of sucrose from 1–3% or more in the upper layer under Fe^{suff} NH_4^+ conditions……”, the statement does not accurately describe the experiment shown in Fig. 7C

Response: The above-mentioned sentence has been revised.

3. Line in methods “clustering and selected for subsequent GO analysis by using R package clusterProfiler.” is an incorrect statement, because R package cannot perform a GO Enrichment Analysis.

Response: We sincerely apologize for omitting the information of the database org.At.tair.db, which provides GO annotations to support the GO term enrichment analysis done by the R package’s clusterProfiler. This information has been added to the revised manuscript (Lines 661-663).

4. Line in methods “GFP/YFP/H2CDFA ex: 488 nm, em:500–550 nm; PI/FM4-64 ex: 561, em: 570–670” need to add a space.

Response: This has been revised.

5. Line in methods “A P <0.05 was considered significant” should be revised.

Response: This has been revised.

6. The scale bar is incorrectly marked in Fig. 1B.

Response: This has been revised.

7. Fig. 3E and 3F: The meaning of the red arrow and green arrow needs to be indicated in the figure legend.

Response: The meanings of the red and green arrows are now indicated in the figure legends.

8. Statistics should be done in Fig. 5F and Fig.5G.

Response: The statistics in Figure 5F and Figure 5G have been performed as suggested.

9. One scale bar is missed in Fig.7B.

Response: The scale bar has been added.

10. Since no Fe deposition was observed in Suppl. Fig. 7, the title “Fe deposition in primary roots of *lpr1lpr2*” of this Figure is not exact.

Response: The words “Fe deposition” in the title have been changed to “Perls/DAB staining” (new Supplementary Fig. 13).

Reviewer #2 (Remarks to the Author):

The manuscript by Liu et al. analyzes a signal mechanism leading to ammonium-induced primary root growth inhibition. They identified a mutant which alleviated ammonium-induced primary root growth inhibition, and which they identified as the ferroxidase LPR2. Its homologue, LPR1 was shown to be crucial player in phosphate deficiency-induced primary root growth inhibition, but a precise role for LPR2 has not been shown yet. In analogy to LPR1, the authors convincingly show, that the cell wall localized LPR2 is involved in ammonium-induced ROS accumulation and callose deposition. They further show that the accumulation of iron, which is the substrate of LPR2, is crucial for ammonium-induced primary root growth inhibition. As such, they provide a model, in which high levels of ammonium triggers apoplastic accumulation of iron, which is oxidized by the action of LPR2, leading to the accumulation of ROS and subsequently to the accumulation of callose. Unlike for the situation in phosphate deficiency, in which these events have been described to occur in the stem cell niche, for ammonium toxicity, these events take place in the differentiation zone of the root, specifically in the phloem. As such, the model is extended by the observation that callose deposition in the phloem impairs its function so that transport of factors with high importance for cellular integrity (such as sucrose) cannot be transported to the lower part of the roots, leading to early differentiation

and primary root growth inhibition.

The results are very interesting and novel and will attract the attention of many scientists working on stress signal transduction. In general, the experiments are properly conducted yielding convincing results, which also justify for most parts the claims by the authors. However, there are some points which should be addressed in order to improve an already good manuscript, which are detailed below.

Response: Thank you very much for your positive comments on our manuscript.

General major comments:

The rescue of ammonium-induced primary root growth inhibition was shown for the mutants *lpr2-1* and *isas*. It is also shown that the WT phenotype can be restored by complementation of the *lpr2-1* mutant by expressing LPR2-YFP under control of the LPR2 promoter. However, all subsequent experiments (iron and callose accumulation, ROS staining) were performed only with *lpr2-1*. It would strengthen the manuscript, if these experiments would be conducted additionally with at least the complementation line as well.

Response: As per your suggestions, we have analyzed Fe deposition, ROS accumulation, callose deposition, phloem transport velocity, and phloem unloading in the complementation line. As expected, all of the measured indexes in the complementation line that are mentioned above behaved similarly to those in Col-0. The respective results have been added to the manuscript. Thank you very much for your valuable suggestions.

Additionally, since the *lpr2-1* mutant can be complemented by a *pLPR2::LPR2-YFP* construct, YFP tagged LPR2 seem to be active. As such, it is reasonable, that in the *p35S::LPR2-GFP* plants, which they used for subcellular localization of LPR2, LPR2 is also active. Therefore, the authors also have generated a stable LPR2 overexpression line. However, nothing beside the subcellular localization of LPR2 is mentioned. It would be interesting to see, if these LPR2 overexpression lines are hypersensitive with respect to ammonium-induced primary root growth inhibition, iron and callose deposition and ROS accumulation. If not, this would mean that

additional factors (such as the discussed availability of the LPR2 substrate Fe) are important factors in the signaling of ammonium-induced primary root growth inhibition.

Response: We found that the complementation lines had higher *LPR2* expression and LPR2 abundance in the roots than those of Col-0 in the NH_4^+-N source with Fe^{suff} (Supplementary Fig. 5), indicating that the *LPR2* was slightly overexpressed in the complementation lines. However, the primary roots of the complementation lines had similar growth inhibition, Fe deposition, ROS burst, and callose accumulation in the NH_4^+-N source with Fe^{suff}, compared to those in the Col-0 seedlings. Therefore, although the NH_4^+ sensitivity of PRs essentially depends on LPR2, we very much agree that additional factor(s) are also important for the phloem-specific Fe deposition and NH_4^+-induced primary root growth inhibition. This notion is also supported by the observation that the region of LPR2 distribution (in all steles except the xylem) is much larger than the site of the heaviest Fe deposition (only in the phloem).

Furthermore, the PRs of *p35S::LPR2-GFP* overexpression lines also had a similar NH_4^+ sensitivity to those of Col-0 (Supplementary Fig. 17a). The results provide further support for the idea that although the NH_4^+ sensitivity of PRs essentially depends on LPR2, additional factor(s) are also required for NH_4^+-induced PR growth inhibition. It is worth noting that the *p35S::LPR2-GFP* overexpression lines showed irregular Fe deposition in PRs in the NH_4^+-N source (Supplementary Fig. 17b), indicating that NH_4^+-induced phloem-specific Fe deposition depends on the stele-specific distribution of LPR2. (lines 238-253)

Furthermore, the *p35S::LPR2-GFP* overexpression lines also showed irregular ROS bursts and callose deposition in PRs in the NH_4^+-N source. Because these irregular distributions of ROS and callose in the PRs of *p35S::LPR2-GFP* overexpression lines do not provide meaningful information for understanding the findings of our study, we did not add those results to our revised manuscript. Thank you very much for your valuable suggestions.

Also, the text should undergo an English spelling and grammar check.

Response: The language of the manuscript has been checked, and modified where necessary, by a professional English editor at Editage (www.editage.cn).

Specific comments:

Introduction: The introduction is very short and not very informative. The authors should mention more about the current state of ammonium toxicity research, i.e. detoxification mechanisms, uptake etc. Also, current knowledge about the signaling events taking place in ammonium-induced primary root growth inhibition should be mentioned, if such information is available.

Response: As per your suggestions, the roles of NH_4^+ uptake and assimilation in NH_4^+ toxicity or detoxification have been mentioned in the Introduction of our revised manuscript (lines 47-55; lines 59-65).

Our current knowledge regarding the signals involved in regulating NH_4^+-induced primary root growth inhibition is very limited, but there are some studies about the signaling events causing NH_4^+ toxicity in shoots. The relevant information has also been added in the Introduction of our revised manuscript (lines 66-73).

Material and Methods:

1. AGI codes should be added to Suppl Table 2 for each gene

Response: The AGI codes have been added (New Supplementary Data 2).

2. Considering the source of the mutants, the authors can already refer to the gene model shown in Figure 1A, in which the location of primers used for genotyping and qRT-PCRs should be indicated as well.

Response: This has been revised as suggested (New Supplementary Fig. 1a). The characterization results of two *lpr2* mutants are shown in Supplementary Fig. 1b-d.

3. Could the authors present a schematic drawing how they measured root meristem size?

Response: As per your suggestion, we have added a photograph illustrating the methods for measuring root meristem size (Supplementary Fig. 3b).

4. Which method was used for calculation of expression values measured by

qRT-PCR?

Response: The respective transcript abundances were determined by normalizing them to the geometric mean of expression of two reference genes for each sample (Vandesompele et al. 2002. *Genome Biol.* 3(7), 1-12). This information has been added to the Material and Methods section (lines 630-631).

5. How was quantification of band intensities for Fig 4B performed?

Response: The LPR2 levels were estimated from the ratio of the signal intensity of LPR2 to that of actin from the same sample. This information has been added to the figure legends.

Results/Discussion:

1. Are the mutants *lpr2-1* and *isas* knock-outs or knock downs? LPR2 Expression levels of all mutants and the complementation and overexpression line should be shown. Also, western blots similar to the one shown in Figure 4B should be shown for both mutants, the complementation and the overexpression lines.

Response: The qPCR assay shows that *LPR2* transcriptions in both *lpr2-1* and *isas* were almost completely abolished under either the NO_3^--N or NH_4^+-N source, although very small amount of PCR product could still be detected (Supplementary Fig. 1). In addition, immunoblot analysis using anti-LPR2 antibodies did not detect a LPR2 signal in *lpr2-1* and *isas*. These results indicate that both *lpr2* lines are most likely knockout mutants.

In Figure 4, the sample of *lpr2-1* from the Fe^{suff} NH_4^+ treatment was used as a negative control in the western blot assays.

The western blots of complementation and overexpression lines have been also added in Supplementary Figure 5 and Supplementary Figure 16, respectively. Surprisingly, although the complementation lines were generated in the background of *lpr2-1*, the detection analysis using anti-LPR2 antibody showed that the signals of untagged LPR2 were also presented in these lines. In addition, the signals of untagged LPR2 in overexpression lines were slightly stronger than those in the Col-0 plants.

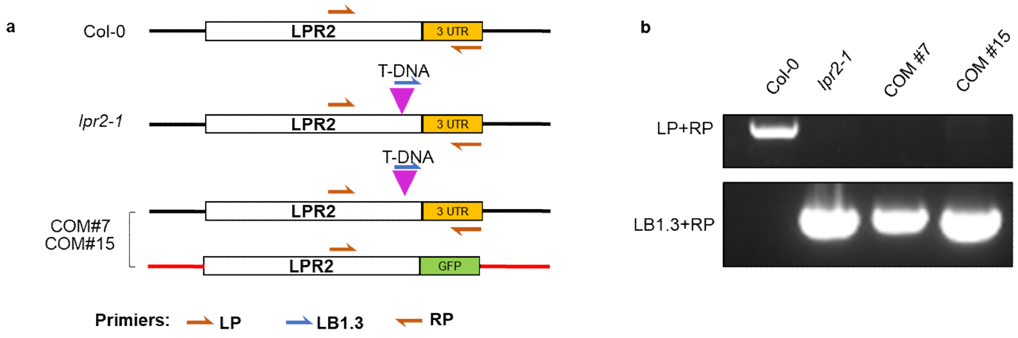

Fig 1 for reviewer: PCR-based genotyping for the genetic background of *pLPR2::LPR2-YFP/lpr2-1* complementation lines. a the primers used for genotyping. **b** the gel picture of PCR-based genotyping.

We first carefully checked whether we had used a wrong background plant for constructing the complementation lines. PCR-based genotyping showed that the complementation lines were indeed constructed in the background of *lpr2-1* (Fig 1 for reviewer). Therefore, the untagged LPR2 was probably caused by the cleavage of LPR2-YFP or LPR2-GFP. The cleavage of GFP from fusion proteins during ER or vesicle trafficking has been described in several studies (Wirth et al., 2007, Journal of Biological Chemistry 282, 23541-23552). In addition, the Col-0 samples showed a weak signal similar to the size of the LPR2-GFP. This signal may be the nonspecific band. To verify the cleavage and nonspecific band in the respective samples, another immunoblot using the Fe^{suff} NH_4^+-treated samples was performed and probed with anti-GFP antibody. Signals at LPR2-GFP/LPR2-YFP size were absent in all Col-0 samples, but present in all over-expression samples. The GFP/YFP signals were also detected in all complementation and overexpression lines (Supplementary Figure 5c and Supplementary Figure 16c). The results confirmed our above speculation. However, the cleavage seems to be much stronger in complementation lines (using the native promoter *pLPR2*) than in overexpression lines (using the ectopic promoter *p35s*). This suggest that the cleavage of GFP/YFP from LPR2-GFP /LPR2-GFP may be tissue specific in plants.

Thank you.

2. Fig 2: Although cell wall localization is plausible according to the picture shown

in Fig 2, the quality of the picture should be improved. There are several examples in the literature, in which plasmolysis was much more evident compared to Figure 2B, which would strengthen the argument that LPR2 is indeed localized to the cell wall. Additionally, are there any hints about the presence of a signal peptide in LPR2 (such as in LPR1)?

Response: Prediction by the SignalP 5.0 algorithm showed that both LPR1 and LPR2 contain a putative secretory signal peptide at the N-terminal region (Supplementary Fig. 5). In addition, we have re-analyzed the subcellular localization of LPR2-GFP in *p35S::LPR2-YFP* transformants using an improved plasmolysis method.

3. Fig 2C and D: There is some confusion in the legend. “Data shown are means ± SD of three biological replicates” refer to Fig 2D, not to Fig 2C.

Response: This has been revised.

4. Fig 2 C and D: It would have been biochemically more relevant, if the GST tag would have been removed. However, this is not a major concern, since the results clearly show that LPR2 is a ferroxidase, which is the major take-home message. However, the authors discuss that (Page 15) “… their enzymatic behaviours are different”. This statement is not valid, since the authors could not show any activity for LPR1. Since native LPR1 has been characterized as a ferroxidase (Reference #20), its activity is probably impaired by the GST tag. Therefore, the authors should refer more to Ref #20 for LPR1 enzyme characteristics and compared it to their LPR2 enzyme characteristics. Also, how were Km and vmax values calculated?

Response: We agree that it would have been biochemically more relevant, if the GST tag was removed. Actually, we tried several times to remove the GST tag from the recombinant proteins using PreScission protease cleavage. Unfortunately, and owing to an unclear problem, the yield of GST-tag-removed LPR2 protein was extremely low, making it difficult to obtain enough protein for the Michaelis–Menten kinetic study. Since the GST-LPR2 had an obvious ferroxidase activity, we used the GST-LPR2 for Michaelis–Menten kinetic study in our work.

Numerous attempts in another study also failed to express active, affinity-tagged

recombinant LPR1 (Naumann et al. bioRxiv, 2021, doi.org/10.1101/2021.03.19.436157). As you suggested, the LPR1 activity was probably impaired by the GST tag; alternatively, the LPR1 protein in *Escherichia coli* may be incorrectly folded. Because the native LPR1 in plants has been convincingly characterized as a ferroxidase (Naumann et al. bioRxiv, 2021, doi.org/10.1101/2021.03.19.436157) and our study focused on the action of LPR2, the failure in detecting ferroxidase activity in recombinant GST-LPR1 did not provide meaningful information for understanding the findings of our study. Therefore, we deleted the result of recombinant LPR1 in the revised manuscript.

The K_m and V_{max} were calculated using the Michaelis–Monod model in the GraphPad Prism 8 software (lines 613-615). Briefly, the V_{max} was defined as the reciprocal of the slope of the Langmuir linear regression equation and the K_m was calculated by multiplying the V_{max} by the intersect of the same equation. In addition, as per your suggestion, we have compared the enzyme characteristics of LPR1 and LPR2 in the Discussion section (lines 428-433). Thank you for your valuable suggestion.

5. Fig 2E: what was the LPR1 expression level in the pLPR2::LPR1 plants? Was it comparable to LPR2 expression levels in WT?

Response: The *LPR1* expression level in the *pLPR2::LPR1* plants has been added in the revised manuscript. As shown in Supplementary Figure 7, the expression of *LPR1* in *pLPR2::LPR1* plants was either slightly higher or lower than that of *LPR2* in Col-0.

6. Fig 3: In contrast to the statement in the last paragraph of page 6, Fe deposition is not shown for *lpr1-1*.

Response: We sincerely apologize for our carelessness. The results of Fe deposition in PRs of *lpr1-1* have been added in Supplementary Figure 13.

7. Is there an explanation for the higher Fe content in the stem cell niche in WT roots exposed to low Fe compared to high Fe?

Response: We are also very surprised at the higher Fe deposition in the root stem cell niche exposed to low Fe, compared to that of high Fe. The RNA-seq result showed that the roots in the low Fe treatment had a higher expression of *FRO2*

(Supplementary Data 1b,d), which encodes a ferric reductase (Robinson et al., Nature, 1999, 397(6721): 694-697). In addition, *FRO2* is preferentially expressed in the root stem cell niche according to the high-throughput single-cell RNA sequencing (Denyer et al., Developmental Cell. 2019, 48(6):840-852). Therefore, a higher *FRO2* expression may favor the provision of more substrate (Fe^{2+}) for ferroxidase to deposit Fe in the stem cell niche.

8. Fig 3 Legend: the abbreviations and the meaning of the arrows should be defined.

Response: This has been revised as suggested.

9. Fig 5, RNA seq results: It is commonly accepted that some expression levels obtained by RNA seq should be confirmed by qRT-PCR. The authors should also provide a table showing the RNA seq data for all genes and not only for the DEGs. Although the intention of the comparisons between high and low Fe is clear, the authors should also provide results from other comparisons, such as between *lpr2-1* and WT, which could be of strong interest to many readers. Also, the data should be discussed in more detail. Are there any differences in expression of genes related to ammonium toxicity (ammonium generation, detoxification, transport etc.) between the treatment/genotypes, especially in relation to the observed root growth phenotypes? The meaning of the scale bar (probably log2 fold) should be added to Fig 5a. What have been the conditions for the results shown in Figure 5E (probably Fe^{suff} NH_4^+)? Page 11: Fig 5 G should read Fig 5 H.

Response: Based on the RNA-seq results, we randomly selected ten genes with different abundances for measuring the expression level through qPCR. As shown in Supplementary Figure 18, the RNA-seq results correlate well with the qPCR results, indicating the reliability of the RNA-seq data. In addition, the RNA-seq data for all genes is also provided (Supplementary Data 1a).

The Fe^{low} treatment and *LPR2* disruption similarly alleviated the NH_4^+-induced PR growth inhibition. Therefore, as per your valuable suggestion, we re-screened the DEGs by performing pairwise comparisons of transcript abundances either between two Fe treatments or between two genotypes. In the NH_4^+-source, there were 182

DEGs (101 upregulated; 81 downregulated) between two Fe treatments for Col-0 and 130 DEGs (58 upregulated; 72 downregulated) between two genotypes for the Fe^{suff} treatment (Supplementary Data 1d, 1e and Supplementary Fig. 19c, d). In these two comparisons, however, we did not find any DEG that is currently known to be related to NH_4^+ toxicity (Supplementary Data 1f). Interestingly, a Venn diagram showed that the ratio of overlapped DEGs was high in the two comparisons mentioned above (Fig. 5a) and gene ontology (GO) enrichment analysis showed that the biggest cluster in both comparisons was related to "response to oxidative stress" (Fig. 5b). (lines 257-277)

Because Venn analysis is more persuasive and informative than Heatmap analysis in explaining the actions of Fe and LPR2, the Heatmap has been replaced by a Venn diagram in the revised manuscript. The condition for Figure 5e has been indicated in the Figures.

10. Fig 6: Although not cell-specific, why does callose overaccumulate in *lpr2-1* mutants? Considering the Fe-ROS-Callose correlation: The authors should discuss why callose accumulation in the stem cell niche of WT exposed to NH_4^+ and Fe low is stronger compared to NO_3^- Fe low, while Fe accumulation is lower in WT exposed to NH_4^+ Fe low compared to NO_3^- Fe low. It is hard to see from the pictures in Fig 6, but is there any preferential accumulation of callose at the sieve plates of the phloem, as has been observed for iron (Fig 3)? This might be important, since it is discussed on page 17.

Response: This is an insightful question. In addition to ROS and Fe deposition, callose accumulation is also affected by other factors. By checking the RNA-seq data (Supplementary Data 1c,e), we found that the expression of *FLS1* (AT5G08640) in *lpr2-1* was clearly inhibited, compared to that in WT in the NO_3^--N source. This may be an explanation for why the PR of *lpr2-1* slightly overaccumulated callose, because the *FLS1* has been shown to negatively affect callose accumulation in plants (Mahajan et al., PLoS one, 2011, 6(12): e28315).

As you mentioned, the callose deposition in the stem cell niche (SCN) did not

coincide with Fe deposition in different treatments. We are also very surprised at this discrepancy. Callose is degraded by β-1,3-glucanases, and bioinformatic analyses have identified approximately 50 β-1,3-glucanase-related genes in *Arabidopsis* (Doxey et al., 2007. Mol. Biol. Evol. 24(4), 1045-1055). A recent single-cell RNA-seq showed that several β-1,3-glucanase-related genes, such as At1g66250, At2g05790, At2g01630 (*PdBG2*), At4g29360, At5g42100, and At5g58090, are preferentially expressed in root SCN (Denyer et al., Dev. Cell. 2019, 48(6):840-852). The impacts of these β-1,3-glucanases in callose accumulation in the SCN may vary in different treatments. If this is true, the impact of β-1,3-glucanases may be an explanation for why the callose deposition in the SCN did not coincide with Fe deposition in different treatments. Relative information has been added to the Discussion section (lines 448-457).

Glycerol was used as the immersion media for root samples to improve the resolution of confocal imaging (Liu and Chiang, Methods. 2019, 30(1), 86-93). By using this method, we found that callose in the phloem was mainly located at the lateral cell walls and sieve plates (Supplementary Fig. 23), which is strikingly similar to the localization of the Fe deposition.

11. Fig 7 D and E: A side by side comparison of the WT response to between NO_3^- and NH_4^+ exposure at different sucrose levels would be more informative. Also, there is only one sucrose concentration (1 %) at which there is a difference, whereas at 2 and 3 %, there is hardly any difference between *lpr2-1* and WT or between NO_3^- and NH4. Did the authors also try even lower sucrose concentrations?

Response: As per your suggestion, we have performed a side by side comparison of the growth response of detached PRs between NO_3^- and NH_4^+ exposure at different sucrose levels (lower sucrose concentrations were also used). In addition, we also tried even lower sucrose concentrations. As shown in Figure 7c, in sucrose-free treatment, the detached PRs almost completely lost the ability to grow, regardless of the N source and genotype, thereby indicating the importance of phloem-transported sucrose in maintaining root growth. For Col-0, the growth difference in detached PRs

between NO_3^-- and NH_4^+-N sources was highly dependent on the dose of sucrose. The difference was the most significant in the 1% sucrose treatment, but it was diminished by reducing sucrose. This is because the growth of detached PRs was inhibited in both N sources. Notably, the inhibitory effect of NH_4^+ on the growth of detached PRs of Col-0 was completely uncoupled by the increase in sucrose to 3% or more. (lines 370-382)

12. Supplemental Figure 11: The meaning of the scale bar should be defined.

Response: The scale bar indicates the emission intensity ratio of 400/485 nm, which shows the oxidation state of roGFP2-Orp1 (new Supplementary Fig. 20). The respective information has been added in the Figure.

Discussion:

The discussion could be more comprehensive. For example: what could be the signaling events downstream of callose deposition leading to ammonium-induced primary root growth inhibition? Could it be that similar to phosphate deficiency, certain factors mediating cell fate, cannot be transported? What about signal molecules etc.?

Response: Thank you for your valuable suggestion. The Discussion has been re-organized. We included more discussions on the difference in mechanisms of Fe deposition between NH_4^+ stress and Pi deficiency (Lines 446-457). In addition, because the impaired phloem action due to excessive callose deposition is responsible for the primary root growth response to NH_4^+ stress, we strengthened the discussion on the role of phloem substances in mediating NH_4^+-induced primary root growth inhibition. In addition to sucrose, the phloem also delivers several other substances, including proteins, RNAs, and peptides. Therefore, we discussed the potential role of these phloem substances in acting downstream of callose deposition, leading to NH_4^+-induced primary root growth inhibition (Lines 485-495).

REVIEWERS' COMMENTS

Reviewer #1 (Remarks to the Author):

Thank you for the clarifications and the additions of new results. The manuscript has been improved significantly. The authors have added additional experiments and answered the questions which I addressed. Now, it is acceptable for publication.

Reviewer #2 (Remarks to the Author):

The manuscript by Liu et al. considerably improved compared to the first version. All my major and minor concerns have been appropriately and comprehensively addressed. There are only a few minor comments, which could be addressed.

1. Material and Methods, Page 26 Line 531: should probably read Supplementary figure 3 (instead of Supplementary figure 2

2. Supp. Fig 10: Both graphs should have the same y-axis range. This would make the effect mor clear

3. Fig 4.e: Please add the genbotypes to the x.axis

4. Page 13, line 262: According to Fig S19 and dataset 1b, there are 111 DEG, not 12 as mentioned in the text

5. DataS1: please indicate in the column header, that log2fold values are shown in addition to the remark at the bottom.

Response to reviewer comments

Reviewer #1 (Remarks to the Author):

Thank you for the clarifications and the additions of new results. The manuscript has been improved significantly. The authors have added additional experiments and answered the questions which I addressed. Now, it is acceptable for publication.

Response: Thank you very much for your positive comments on our manuscript.

Reviewer #2 (Remarks to the Author):

The manuscript by Liu et al. considerably improved compared to the first version. All my major and minor concerns have been appropriately and comprehensively addressed. There are only a few minor comments, which could be addressed.

Response: Thank you very much for your positive comments on our manuscript.

1. Material and Methods, Page 26 Line 531: should probably read Supplementary figure 3 (instead of Supplementary figure 2)

Response: This has been corrected.

2. Supp. Fig 10: Both graphs should have the same y-axis range. This would make the effect more clear

Response: The necessary change has been carried out.

3. Fig 4.e: Please add the genotypes to the x.axis

Response: The genotypes have been added.

4. Page 13, line 262: According to Fig S19 and dataset 1b, there are 11 DEGs, not 12 as mentioned in the text

Response: Thanks for pointing this out. We have corrected the number of DEGs.

5. Data S1: please indicate in the column header, that log2fold values are shown in addition to the remark at the bottom.

Response: The suggested change has been incorporated.
